# Automated On-the-Fly Optimization of Resource Allocation for Efficient Free Energy Simulations

**DOI:** 10.1021/acs.jcim.4c02107

**Published:** 2025-05-06

**Authors:** S. Benjamin Koby, Evgeny Gutkin, Shree Patel, Maria G. Kurnikova

**Affiliations:** Department of Chemistry, 6612 Carnegie Mellon University, Pittsburgh, Pennsylvania 15213, United States

## Abstract

Computing the free energy of protein–ligand binding by employing molecular dynamics (MD) simulations is becoming a valuable tool in the early stages of drug discovery. However, the cost and complexity of such simulations are often prohibitive for high-throughput studies. We present an automated workflow for the thermodynamic integration scheme with the “on-the-fly” optimization of computational resource allocation for each λ-window of both relative and absolute binding free energy simulations. This iterative workflow utilizes automatic equilibration detection and convergence testing via the Jensen–Shannon distance to determine optimal simulation stopping points in an entirely data-driven manner. It is broadly applicable to multiple free energy calculations, such as ligand binding, amino acid mutations, and others, while utilizing different estimators, e.g., free energy perturbation, BAR, MBAR, etc. We benchmark our workflow on the well-characterized systems, namely, cyclin-dependent kinase 2 and T4 lysozyme L99A/M102Q mutant, and the more flexible SARS-CoV-2 papain-like protease. We demonstrate that this proposed protocol can achieve more than 85% reduction in computational expense while maintaining similar levels of accuracy compared to other benchmarking protocols. We examine the performance of this protocol on both small and large molecular transformations. The cost–accuracy tradeoff of repeated runs is also investigated.

## Graphical abstract

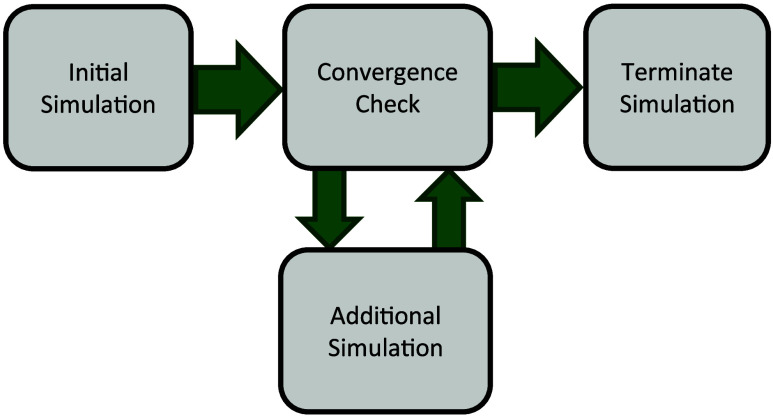

## Introduction

Free energy differences in biomolecular systems are often required to quantitatively characterize the structure and dynamics of biomolecular interactions underlying multiple biological and biochemical processes.^1^ One such important quantity is the binding free energy (BFE) of a small-molecule ligand to a protein.^2,3^

BFEs can be categorized into two broad classes: absolute BFE (ABFE) and relative BFE (RBFE). ABFE is the difference in free energy between the protein–ligand complex and the free protein and ligand in solution, referred to as the standard binding free energy, Δ*G*_bind_^o^. This quantity can be measured experimentally, e.g., by surface plasmon resonance^4^ or isothermal titration calorimetry.^5^ Δ*G*_bind_^o^ is related to the ligand dissociation constant *K*_d_ by the following relation

1 $$\Delta G_{\mathrm{bind}}^{{}^{\circ}}=\mathrm{RT}\ln K_{d}$$

where *R* is the gas constant and *T* is the temperature of the system. RBFE is the difference in Δ*G*_bind_^o^ of two ligands A and B, referred to as ΔΔ*G*_A→B_^bind^. In drug design applications, ABFE simulations can be used for initial screening and hit identification of compounds for synthesis,^6,7^ while RBFE simulations are well suited for hit-to-lead and lead optimization.^8–12^

A common approach to computing BFE is molecular dynamics (MD)-based thermodynamic integration^13^ (TI) that utilizes an “alchemical”—i.e., nonphysical—thermodynamic pathway. MD TI and other types of simulations^14,15^ that utilize an alchemical thermodynamic pathway to compute BFE are often termed alchemical binding free energy calculations. MD TI can be used to compute both ABFE and RBFE; however, the two methods have several technological differences in implementation, mainly in the alchemical pathways employed. MD TI simulations are expensive and technologically complex, and historically, scaling up these computations to include multiple ligands has been difficult.

With the advent of GPU computing, MD TI simulations have become feasible for high-throughput drug discovery applications;^7,8,16–21^ however, computing BFEs for a large number of ligands remains technically difficult, costly, and time-consuming.^22^ The approach suffers from various technical difficulties, including tedious system setup and preparation; inaccuracies in modeling difficult transformations such as ring breaking/forming, ring extensions, and changes in the net charge;^12,23–26^ and the difficulty in achieving sufficient sampling across all intermediate states. Combined, these problems often prove unsurmountable, leading to the selection of cheaper and simpler methods with inferior accuracy. Simplified computational methods, e.g., molecular docking, a commonly used computational screening technique, can quickly yield reasonable ligand binding poses; however, its scoring functions are typically insufficiently accurate for discriminating false positives from true hits,^6,7,27–29^ which is a serious problem when thousands of potential hits have been proposed with limited resources for experimental validation. Endpoint MD methods, such as MM/PBSA and MM/GBSA, may sometimes afford limited accuracy improvement compared to docking, albeit at a greater cost; yet their accuracy is strongly system-specific and is generally less reliable than MD TI simulations.^30,31^

Recently, we have developed workflows for high-throughput ABFE and RBFE calculations for small molecules.^7,8^ A hit-to-lead optimization framework combining RBFE simulations and active learning (AL) was designed to iteratively explore large chemical spaces consisting of thousands of congeneric molecules for high-performing molecules compared to an experimentally validated reference compound.^8,20^ In this scheme, batches of molecules are iteratively selected by a machine learning (ML) model trained on previously performed RBFE calculations and a featurization of the respective molecules, with an overall goal of harvesting the molecules with the most negative ΔΔ*G*_A→B_^bind^ value. In initial iterations, molecules are sampled in an exploratory manner, focusing on areas of the chemical space where the ML model is most uncertain. Latter iterations sample in an exploitative fashion, where the molecules selected are predicted to have the most negative ΔΔ*G*_A→B_^bind^ value. After RBFE calculations are performed on a sample, it is used to augment the training data for further AL cycle iterations. With this framework, we were able to explore a chemical space consisting of 8715 ligands with only 253 simulations, identifying 133 compounds with a higher predicted affinity. Others have also demonstrated the active learning framework efficacy in RBFE simulations,^32–34^ structural docking screening,^35–37^ force field development,^38^ and coarse graining.^39^ Even with our active learning framework, the computational cost of requisite simulations requires significant resources, often in the thousands of GPU hours.^8,20,34^

ABFE simulations have been proposed as an accurate and cost-effective method for the final stages of high-throughput virtual drug screening for initial hit discovery.^6^ Beginning with large data sets of molecules, often in the millions to billions, this approach utilizes high-throughput docking to narrow down the set of candidates to a number feasible for ABFE simulations, typically in the hundreds or thousands, with top-performing candidates submitted for experimental validation.^6,7,28^ The utility of combining docking with alchemical BFE simulations has been recognized for several decades due to their complementary nature: the inaccuracy of docking can be corrected with ABFE rescoring, while docking both significantly reduces the number of ABFE simulations required and provides reasonable starting poses.^40^ Still, this may involve thousands of tedious and expensive calculations, which can be infeasible to perform. Thus, both RBFE and ABFE simulations would benefit from methods designed to decrease the cost of such high-throughput computations.

Here, we present a simple, highly automated, and data-driven approach for on-the-fly optimization of computational resource allocation for high-throughput TI RBFE and TI ABFE simulations. The goal of this protocol is to utilize the minimal resources necessary to achieve a convergence with the desired accuracy for each individual simulation with as little user input as possible. We begin by describing the theory behind alchemical BFE simulations and the simulation parameters employed for each system studied in this work. Next, we illustrate the workflow and concepts behind our on-the-fly optimization algorithm. We then demonstrate our RBFE implementation on the cyclin-dependent kinase 2 (CDK2) benchmark system using the same set of ligands as Song et al.^41^ and compare our accuracy with respect to experimental results against theirs. In order to examine the performance of our protocol on more difficult and flexible systems, we apply several implementations of our protocol to several ligand mutations of the SARS-CoV-2 papain-like protease (PLpro), which we have performed in previous work.^8^ As PLpro is less characterized than CDK2, we compare our results against long simulations as opposed to experimental results. Next, we apply our ABFE implementations to compute the affinity of the T4 lysozyme L99A/M102Q mutant to *N*-phenylglycinonitrile (PDB ID: 2RBN^42^) and compare the accuracy with respect to experimental results against that of a protocol we utilized in our previous work.^7^ We also compare several optimized protocol implementations on the PLpro ligand *N*-[(1*R*)-1-naphthalen-1-ylethyl]­benzamide against long simulations. We demonstrate that our protocol maintains accuracy while yielding increases in computational efficiency compared to previously published sampling schemes. Finally, we conclude by making prescriptions for future high-throughput alchemical BFE drug discovery campaigns.

## Methods

### Thermodynamic Integration Molecular Dynamics Simulations

Thermodynamic Integration is a standard technique, which is briefly described below for completeness. MD TI BFE simulations are designed to compute the relative free energy of two molecular systems by transforming one molecular system into another by performing multiple stratified equilibrium MD simulations along a predefined reaction coordinate, i.e., an alchemical thermodynamic pathway. Typically, the pathway is defined as a linear interpolation between the Hamiltonians of two systems, A and B

2 $$V\left(\lambda \right)=\lambda V_{A}+\left(1-\lambda \right)V_{B}$$

where *V*(λ) is the coupling potential energy function, λ ∈ [0,1] defines the coupling parameter, and *V*_A/B_ is the potential energy of the endpoint A or B, respectively. The free energy difference between endpoints A and B, Δ*G*_A→B_, is derived by integrating the derivative of the coupling potential energy function with respect to λ

3 $$\Delta G_{A\to B}=\int_{0}^{1}{\left\langle \frac{dV}{d\lambda }\right\rangle }_{\lambda }d\lambda ≅\sum_{1}^{N}w_{i}{\left\langle \frac{dV}{d\lambda }\right\rangle }_{i}$$

where ${\left\langle \frac{dV}{d\lambda }\right\rangle }_{\lambda }$ is the average of the derivative of the coupling potential energy function (further referred to as the gradient time series for simplicity) assuming continuous λ. In practice, the integral is performed numerically using a discrete set {λ_*i*_}_1_^N^, where ${\left\langle \frac{dV}{d\lambda }\right\rangle }_{i}$ is the average of the gradient time series of the λ_i_-window MD simulation and *w*_*i*_ is the statistical weight of the strata determined by the selected integration scheme.

Usually, in a single replica scheme, each MD simulation involved in an alchemical transformation is independent of the others. Some simulation schemes, however, do not employ independent simulation schemes such as those employed by He et al.,^16^ where initial structures for a simulation were obtained from snapshots of simulations from neighboring strata. Multiple replica schemes, including replica exchange^43^ and Hamiltonian exchange variants like REST,^44,45^ are also utilized in the field. These schemes may increase the rate of convergence for some systems but suffer from known issues derived from the inherent tradeoff between an enhanced sampling state and the efficient exchange of replicates.^46–48^ In some systems, the use of replica exchange algorithms may lead to inferior results.^49^

Another class of methods, nonequilibrium work methods,^50–52^ utilize swarms of short simulations in which λ is driven from one extreme to the other. The free energy change of the mutation can then be calculated as a function of nonequilibrium work. While promising for driving down the cost of computing binding free energies, these methods have not been widely adopted in the field.

### Relative Binding Free Energy Thermodynamic Cycle

The thermodynamic cycle for a TI RBFE simulation is depicted in Figure 1A. The difference in Δ*G*_bind_^o^ between ligands A and B, ΔΔ*G*_bind_^A→B^, is computed by performing an alchemical mutation of the ligands from one to the other in both the bound and unbound states

4 $$\Delta \Delta G_{\mathrm{bind}}^{A\to B}=\Delta G_{\mathrm{bind}}^{A}-\Delta G_{\mathrm{bind}}^{B}=\Delta G_{A\to B}^{\mathrm{prot}}-\Delta G_{A\to B}^{\mathrm{wat}}$$

where Δ*G*_bind_^A/B^ is the absolute binding free energy of ligands A/B, Δ*G*_A→B_^prot^ is the change in free energy of mutating ligand A into ligand B in complex with the protein, and Δ*G*_A→B_^wat^ is the change in free energy of mutating ligand A into ligand B in solution (Figure 1A). Thus, TI RBFE calculations consist of two alchemical transformations: from one ligand to another while in complex with the protein and from one ligand to another while in solution. While in principle any mutation could be accomplished, only minor mutations are commonly performed due to the rapid accumulation of errors in large transformations.^8–10,20,41^ Therefore, RBFE simulations are typically restricted to sets of congeneric ligands that share a common substructure.

**Figure 1:**
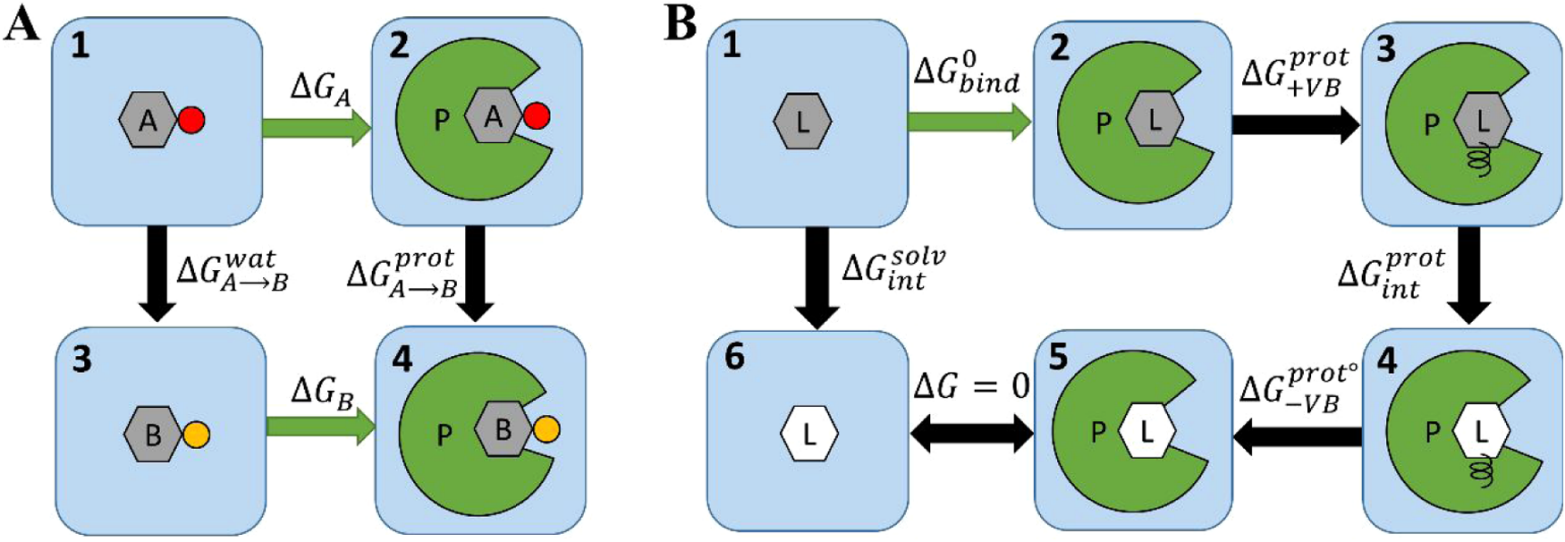
Alchemical thermodynamic cycles for calculation of (A) RBFE, corresponding to eq 4, which consists of two alchemical transformations, and (B) ABFE, corresponding to eq 5, which consists of three alchemical transformations. The arrows determine the direction of the transformation and therefore the sign of the term in the sum in eqs 4 and 5.

### Absolute Binding Free Energy Thermodynamic Cycle

The thermodynamic cycle for an ABFE MD simulation is shown in Figure 1B. The free energy difference between the protein–ligand complex and the free protein and ligand in solution (Figure 1B 1 → 2: Δ*G*_bind_^o^) is calculated by completing an alchemical pathway between these two endpoint states utilizing a virtual bond following the approach of Boresch et al.^53^ (details described in the Methods section and the SI).

5 $$\Delta G_{\mathrm{bind}}^{{}^{\circ}}=\Delta G_{\mathrm{int}}^{\mathrm{solv}}-\Delta G_{+\mathrm{VB}}^{\mathrm{prot}}-\Delta G_{\mathrm{int}}^{\mathrm{prot}}-\Delta G_{-\mathrm{VB}}^{\mathrm{prot}{}^{\circ}}$$

where Δ*G*_int_^solv^ is the free energy of the removal of the electrostatic and van der Waals interactions, often termed “annihilation” of the ligand, between the ligand and the environment while in solution (Figure 1B: 1 → 6); Δ*G*_+VB_^prot^ is the free energy of the addition of a virtual bond^53^ between the protein and the ligand in the protein–ligand complex simulation (Figure 1B: 2 → 3, see Gutkin et al. for details); Δ*G*_int_^prot^ is the free energy of the annihilation of the ligand in complex with the protein and while restrained by the virtual bond (Figure 1B: 3 → 4); and Δ*G*_‑VB_^prot°^ is the free energy of removing the virtual bond from the annihilated ligand, which is computed analytically (Figure 1B: 4 → 5).^53^ There is no free energy cost to remove the ligand from the binding pocket in the last step in Figure 1B (5 → 6).

#### Software

All MD simulations were performed using the GPU-accelerated pmemd.cuda module of AMBER20.^54–57^ All $\frac{dV}{d\lambda }$ gradient time series data (eq 3) were extracted with the alchemlyb^58^ Python package. Decorrelation was performed using the pymbar^59^ Python package time series analysis module, whereby decorrelated samples were obtained by subsampling with the statistical inefficiency rounded up to the nearest integer value. All hydrogen-bonding interactions between the protein and ligand were analyzed with CPPTRAJ.^60^ All input coordinates, topologies, and parameters for conventional MD simulations were obtained using Ambertools18.^61^

### Molecular System Simulations

#### RBFE CDK2 System Setup and TI Simulations

Simulation starting structures for protein–ligand complexes were extracted from the GitHub repository (https://github.com/linfranksong/Input_TI.41). For both the protein–ligand complex and solvated ligand mutations, two separate λ-schedules were used. The first employed the following 12 λ-windows: 0.00922, 0.04794, 0.11505, 0.20634, 0.31608, 0.43738, 0.56262, 0.68392, 0.79366, 0.88495, 0.95206, and 0.99078. The second employed the following nine λ-windows: 0.01592, 0.08198, 0.19331, 0.33787, 0.5, 0.66213, 0.80669, 0.91802, and 0.98408. For each λ-window, the following protocol was employed: (1) 2000 steps of minimization with the gradient descent method, (2) 50 ps of heating from 0.1 to 300 K in the NVT ensemble, (3) 300 ps of density equilibration in NPT, and (4) production simulations in NVT with gradient averages obtained via the bootstrap method. Harmonic RMSD restraints were imposed on heavy atoms of the protein and the ligand during minimization and heating and were gradually removed during density equilibration; no restraints were used during production simulations. Special care was given to the λ = 0.98408 window of the nine-point λ-schedule to avoid numerical instability. For this window, the structure obtained after the first 1000 steps of minimization with gradient descent of the λ = 0.91802 window was used as the input for the second 1000 steps of minimization, and then the protocol proceeded as normal. Softcore potentials were utilized for all simulations. For the 12-window λ-schedule production simulations, which were implemented to closely mimic the production protocol of Song et al., a 1 fs time step was used without SHAKE^62^ and a Berendsen thermostat^63^ was employed. All other protocols utilized a Langevin thermostat. For the nine-point λ-schedule, which was implemented according to our standard practices, a 2 fs time step was used with SHAKE. Free energies of both the protein–ligand complex and solvated ligand alchemical steps were obtained using the Gaussian quadrature rule. For each mutation, a total of ten independent simulations were performed per protocol. All uncertainties were quantified using samples of multiple replicates.

#### RBFE PLpro System Setup and TI Simulations

Four ligands were selected from our previous work on the PLpro system^8^ and are displayed in Figure 2. Ligands 1–3 were selected such that their ΔΔ*G*_A→B_^bind^ values calculated in previous work were negative (ligand 1), approximately 0 kcal/mol (ligand 2), and positive (ligand 3). Ligand 4 was selected as an edge case due to the difficulty of the mutation (see the Results and Discussion section). Input coordinates, topologies, and parameters were obtained from our previous work (see Gusev et al.^8^ for details). All λ-windows were minimized and equilibrated using the protocol described in the previous section, with restraints applied to two water molecules in the binding pocket during minimization and heating and gradually removed during density equilibration. For each mutation, ten replicates were performed per protocol. All uncertainties were quantified using samples of multiple replicates.

**Figure 2:**
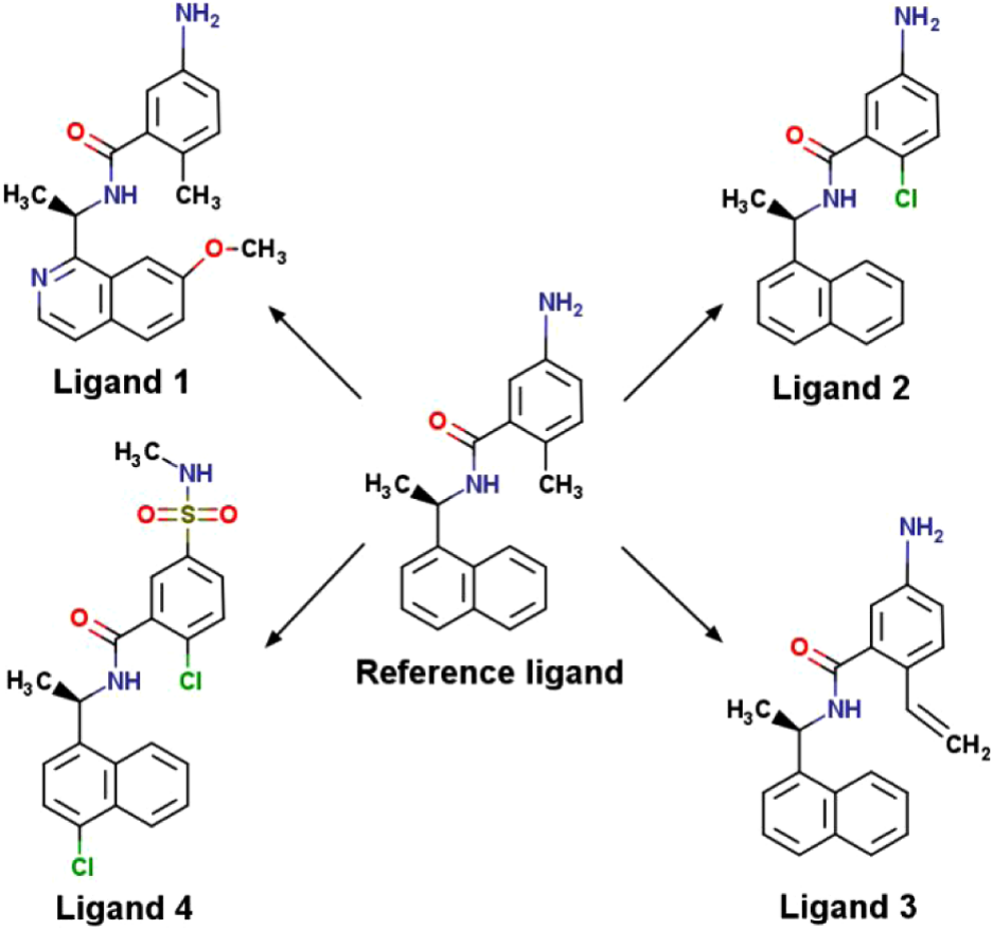
PLpro ligands used for ABFE and RBFE simulations. ABFE simulations were performed on the reference ligand, while RBFE simulations were performed by mutating the reference ligand to ligand 1, 2, 3, or 4.

#### ABFE Lysozyme Protein System Preparation and Simulations

The crystal structure of the T4 lysozyme L99A/M102Q in complex with *N*-phenylglycinonitrile was extracted from the Protein Data Bank (PDB ID: 2RBN^42^). Ligand atom parameters were obtained using GAFF2 (version 2.11),^61^ and ligand atomic charges were derived using the RESP^64^ method with Gaussian 09 ^65^ or 16.^66^ The protein was parametrized with the FF14SB force field solvated in an orthorhombic TIP3P^67^ water box using tleap with a 15 Å distance between the protein and the edge of the box. The simulation protocol included the following steps: (1) 2000 steps of minimization with the gradient descent method, (2) 100 ps of heating from 1 to 300 K in the NVT ensemble, (3) 300 ps of density equilibration in the NPT ensemble, and (4) 7 ns of production simulation in NVT. All MD simulations were performed using a 2 fs time step. Harmonic RMSD restraints were imposed on heavy atoms of the protein and the ligand during minimization and heating and were gradually removed during density equilibration; no restraints were used during production simulations. The first 2 ns of the MD production simulation were discarded, and the average structure was obtained from the last 5 ns of the simulation. A trajectory frame with a minimum RMSD of ligand heavy atoms with respect to the average structure was selected as a representative structure and used as the initial protein–ligand complex structure for TI simulations. A total of 39 independent replicates were performed.

#### ABFE Lysozyme TI Simulations

For TI simulations of the solvated ligand, the ligand was solvated in a TIP3P water box using tleap^61^ with a 15 Å distance between the ligand and the edge of the box. For TI simulations of the protein–ligand complex, the orientation of the ligand with respect to the protein was restrained using the virtual bond approach (see the SI for details regarding the selection of atoms for the virtual bond).^29,53^ Force constants of 4 kcal/(mol·Å^2^), 20 kcal/(mol·rad^2^), and 40 kcal/(mol·rad^2^) were used for distance, angle, and dihedral angle restraints, respectively. The second-order smoothstep softcore potential (SSC(2)), as implemented in AMBER20, was utilized for both the protein–ligand complex and solvated ligand steps. For each λ-window, the system was minimized and then equilibrated using the same protocol as was performed for conventional MD. For the solvated ligand and protein–ligand complex systems, a λ-schedule of nine equally distributed windows was used (0.1, 0.2, 0.3, ···, 0.9). Gradient means and variances were calculated using the bootstrap method.^68^ For the addition of the virtual bond restraints, seven unequally distributed λ-windows were used (0.0, 0.05, 0.1, 0.2, 0.3, 0.5, 1.0). Free energies for the ligand, protein, and restraint addition were obtained via the trapezoid rule. The free energy of adding virtual bond restraints for the noninteracting ligand was calculated using the Boresch formula.^53^ A total of 39 independent simulations were performed for all protocols. All uncertainties were quantified using samples of multiple replicates.

#### ABFE PLpro System Setup and TI Simulations

ABFE simulations were performed on *N*-[(1*R*)-1-naphthalen-1-ylethyl]­benzamide, henceforth referred to as the “reference ligand.” Input coordinates, topologies, and parameters were obtained from our previous work^8^ (see Gusev et al. for details). Force constants of 10 kcal/(mol·Å^2^), 10 kcal/(mol·rad^2^), and 20 kcal/(mol·rad^2^) were used for distance, angle, and dihedral angle restraints, respectively. All λ-windows were minimized and equilibrated using the protocol described above, with the addition of harmonic restraints applied to two water molecules in the binding pocket during minimization and heating, and these restraints were gradually removed during equilibration. No restraints were applied during production. A total of 30 independent simulations were performed for all protocols. All uncertainties were quantified using samples of multiple replicates.

### Approach

It is typical to uniformly allocate computational resources by simulating each λ-window for the same amount of simulation time;^7,8,10,16,21,41,69^ however, there is no theoretical basis for this practice. For instance, it is unlikely that simulation times necessary for convergence would be equivalent between different systems, such as the protein–ligand complex and the solvated ligand, even if we restrict our analysis to states with identical λ-values. One would expect this likelihood to decline further when considering different ligands or even different protein systems. Furthermore, it is not uncommon for specific λ-windows of a given alchemical transformation to experience larger autocorrelation times than other λ-windows, resulting in slower convergence, fewer uncorrelated samples, and greater statistical uncertainty. In contrast, other λ-windows converge very quickly, with additional sampling affording marginal accuracy benefits at best. In short, using a uniform allocation of resources may result in wasting resources simulating already converged λ-windows while starving more difficult λ-windows of resources. This issue is magnified when considering a high-throughput computational drug discovery campaign due to the large number of necessary simulations. Below, we propose an algorithm to address this problem in an automatic and entirely data-driven manner without a priori information on the system in question.

#### On-the-fly Optimization Algorithm

We developed and tested an algorithm for the on-the-fly optimization of computational resources used by MD TI simulations to predict small-molecule binding energies, as shown in Figure 3: Starting from the equilibrated structure, an initial short production simulation was performed and the $\frac{dV}{d\lambda }$ gradient time series (eq 3) was extracted. Convergence testing to determine whether the production simulation should be extended was performed as follows. The $\frac{dV}{d\lambda }$ gradient time series was equilibrated with automatic equilibration detection^70^ (AED), as implemented in the pymbar Python package time series analysis module. This method determines the optimal equilibration time as the point that maximizes the uncorrelated sample size obtainable from an equilibrated gradient time series. After equilibration, the $\frac{dV}{d\lambda }$ gradient time series was decorrelated (see the Methods section) and then split in half chronologically, with each half binned into seven equally spaced bins. The Jensen–Shannon (JS) distance,^71^ a measure of distance between two probability distributions, between these two histograms was calculated. Given two probability distributions *P* and *Q*, the JS distance is defined as follows

6 $$\mathrm{JS}(P|\left|Q\right)=\sqrt{\frac{1}{2}(D(P|\left|M\right)+D\left(Q|\left|M\right)\right)}$$

where *D*(*P*||*M*) is the Kullback–Leibler divergence and $M=\frac{1}{2}\left(P+Q\right)$ . The JS distance between the two histograms was then used as the convergence criterion: if the JS distance was less than or equal to 0.1, roughly the tenth percentile of similarity between the two histograms, the simulation was terminated. If the JS distance was greater than or equal to 0.1 or the total number of decorrelated samples was fewer than 50 (see below for explanation), the production simulation was extended for a predefined time. This was repeated until convergence or until either of the following two scenarios were met: a total simulation time of 6.5 ns was achieved and more than 50 decorrelated samples were acquired in total, or a total simulation time of 10.5 ns was achieved (Figure 3).

**Figure 3:**
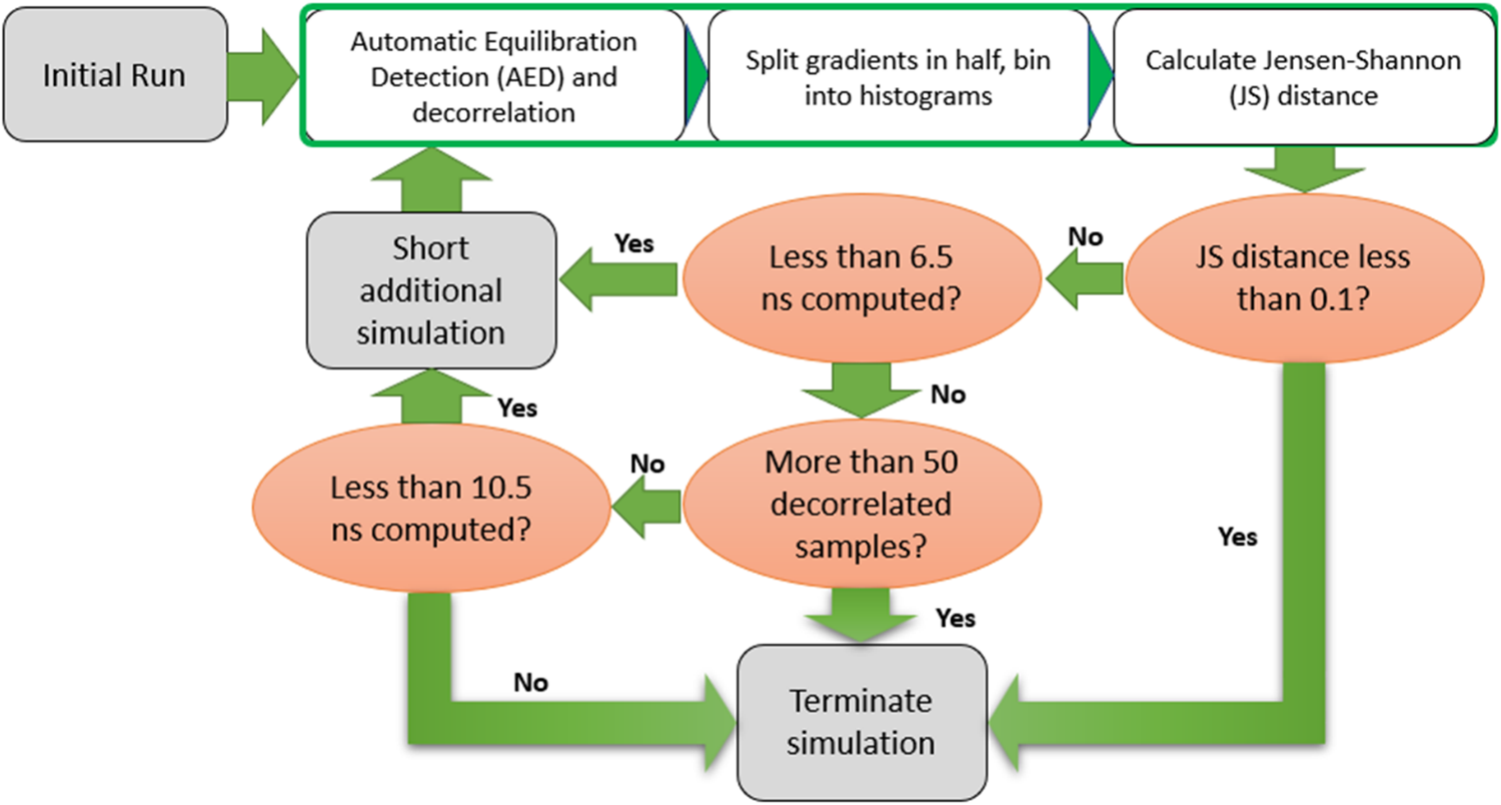
Flowchart of the on-the-fly resource optimization for high-throughput binding free energy MD TI simulations. This protocol is applied to each individual λ-window.

The underlying idea behind the proposed algorithm is that the amount of simulation time needed to converge the gradient time series is difficult to predict a priori; thus, in order to ensure the application of the minimally necessary amount of simulation time, one must frequently check for convergence in an entirely automated fashion. Multiple contradictory objectives are balanced by the choice of several hyperparameters. The potentially fractal nature of the protein dynamics implies that some simulations exhibit behavior that may not be sampled at short times. The observation of such behavior is controlled by the initial simulation length hyperparameter. The frequency of convergence checking is controlled by the additional simulation length hyperparameter. The probability of the gradient time series being observed as converged at any point in the simulation is nonzero. Thus, more frequent convergence testing will result in a higher likelihood of a false positive convergence. The JS distance convergence threshold hyperparameter has a similar effect: higher values increase the likelihood of obtaining faster convergence and false positives because the two histograms need lower degrees of similarity to meet this threshold, while lower values decrease this probability but also may cause unnecessarily longer simulations. We chose to use at least 50 decorrelated samples to be comfortably above the rule of thumb of sample sizes in standard statistical analysis but not too high to frequently force additional sampling for marginal benefit because the uncertainty will decrease with the inverse square of the sample size. Furthermore, Clark et al. suggested that due to long autocorrelation times, reductions in statistical uncertainty often do not scale with the amount of sampling. Thus, increasing this requirement will yield diminishing returns as the sample size grows, assuming that the simulation is efficiently sampling the relevant configurations.

The number of histogram bins also affects the estimated rate of convergence for a given sample size: larger numbers of bins will magnify differences between the distributions, effectively making them noisier for a given number of decorrelated samples^72^ and yielding a stricter convergence criterion, whereas smaller numbers of bins will blur these differences and accelerate convergence acceptance. If one requires a minimum of more decorrelated samples than our current threshold of 50, the number of bins could be increased; however, we note that others have found diminishing statistical accuracy with increased sampling^73^ due to the tendency of these simulations to inefficiently sample the entire ensemble of configurations, leading to large autocorrelation times and therefore resulting in expensive acquisition of decorrelated samples. Finally, some simulations may require large amounts of simulation time to obtain convergence, which may be greater than the computational budget allows. Balancing between the computational budget and allowance for long simulations is controlled by the maximum simulation length hyperparameters, which should be adjusted according to one’s computational budget. In instances when convergence is difficult to achieve in the budgeted simulation length, more advanced methods such as Hamiltonian exchange variants^10,46,69^ may be necessary; however, their evaluation is beyond the scope of this work. We note that our method is applicable to any application of stratified time series analysis beyond protein–ligand binding free energy simulations, including solvation free energy, amino acid mutations, and protein–protein binding free energy.

While the standard in the field is to apply uniform sampling time to all windows, additional sampling time may occasionally be added based on various convergence criteria, typically functions of the window error estimates from either TI or BAR utilizing a single^74–76^ or multiple^73,77,78^ replicates. Our method differs in that we measure convergence based on the distribution of the gradient time series without explicitly computing the variances of or between various windows. For TI, the overall variance of a single calculation often greatly underestimates the variance observed from multiple runs; thus, utilizing metrics independent of the variance of the calculation may yield more reliable results. The fact that the variance of a window may not correctly reflect the accuracy of the simulation has been noted by others.^73^ We plan to address this issue in future work. Furthermore, not requiring the use of multiple replicates streamlines and simplifies the overall workflow while still allowing for the use of repeated runs if resources allow. This is important in a high-throughput setting when hundreds or thousands of individual free energies are to be computed in a limited amount of time. We note that a complementary strategy is to optimize the λ-schedule to increase efficiency and accuracy. Several methods^47,73,79,80^ have been developed to these ends, typically involving a priori information obtained from a short burn-in run of a default λ-schedule before an optimized one may be selected.

## Results and Discussion

The on-the-fly simulation optimization approach described above was implemented in protocols for both RBFE and ABFE calculations and tested using three protein systems: CDK2 (RBFE), T4 lysozyme L99A/M102Q mutant (ABFE), and PLpro (RBFE and ABFE). CDK2 and Lysozyme are common benchmarking systems, allowing us to compare the results from our optimized protocols against published alternatives.^7,41^ The PLpro protein is a more flexible and less well characterized system, thus offering a more difficult challenge for both ABFE and RBFE. For this system, we compared our optimized protocols to long-run protocols ranging from 10 to 100 ns per λ-window. In total, we evaluated six different implementations of our algorithm: protocols A–E and C12, as summarized in Table 1.

**Table 1:** Parameters of the Various On-the-Fly Optimization Protocols Employed

protocol	initial simulation length (ns)	additional simulation length (ns)	number of λ-windows
*A*	2.5	0.5	9
*B*	1.5	0.5	9
*C*	1.0	0.25	9
*D*	0.5	0.25	9
*E*	3.5	0.5	9
*C12*	1.0	0.25	12

Protocols A–E differ solely in the amount of simulation time used in the initial and additional simulation steps. Protocol A uses a 2.5 ns initial simulation with 0.5 ns additional simulations, protocol B uses a 1.5 ns initial simulation with 0.5 ns additional simulations, protocol C uses a 1.0 ns initial simulation with 0.25 ns additional simulations, protocol D uses a 0.5 ns initial simulation with 0.25 ns additional simulations, and protocol E uses a 3.5 ns initial simulation with 0.5 ns additional simulations. Protocol C12 was only employed for RBFE calculations for CDK2 and utilized the 12-window λ-schedule with a 1.0 ns initial simulation and 0.25 ns additional simulations (see the Methods section for further details on the differences between the protocols).

### RBFE Calculations for CDK2

Experimental ΔΔ*G*_A→B_^bind^ values for each mutation were obtained from Song et al.^41^ The overall mean absolute error (MAE) and root-mean-squared error (RMSE) between experimental and computed ΔΔ*G*_A→B_^bind^ values, when considering all ten replicates per mutation, were 1.03 and 1.24 kcal/mol for protocol A, 1.02 and 1.29 kcal/mol for protocol B, 0.99 and 1.25 kcal/mol for protocol C, and 0.96 and 1.24 kcal/mol for protocol C12 (see Table 1 for protocol details). The overall *R* ^2^ values for protocols A, B, C, and C12 were 0.26, 0.23, 0.28, and 0.30, respectively. A permutation test was performed, whereby 10,000 MAEs and RMSEs were computed by randomly permuting the experimental ΔΔ*G*_A→B_^bind^ values. For all protocols and both MAE and RMSE, the calculated values were less than 99% of these generated values, which indicates that there is a significant association between our predicted ΔΔ*G*_A→B_^bind^s and their experimental values. This is important as the null model (ΔΔ*G*_A→B_^bind^ = 0 for each mutation) performs extremely well on this system, as others have noted,^41^ with the MAE and RMSE of 0.95 and 1.23 kcal/mol, respectively. Figure 4 displays the simulated distributions of MAE, RMSE, and *R* ^2^ of protocol A with respect to the number of replicates, referred to hereafter as batch size, included in the calculation. These distributions were generated by taking 10,000 random combinations of the given sample size of replicates, averaging the ΔΔ*G*_A→B_^bind^ and then calculating the MAE, RMSE, and *R* ^2^, respectively. The tabulated summary statistics for these distributions are given in Table S1.

**Figure 4:**
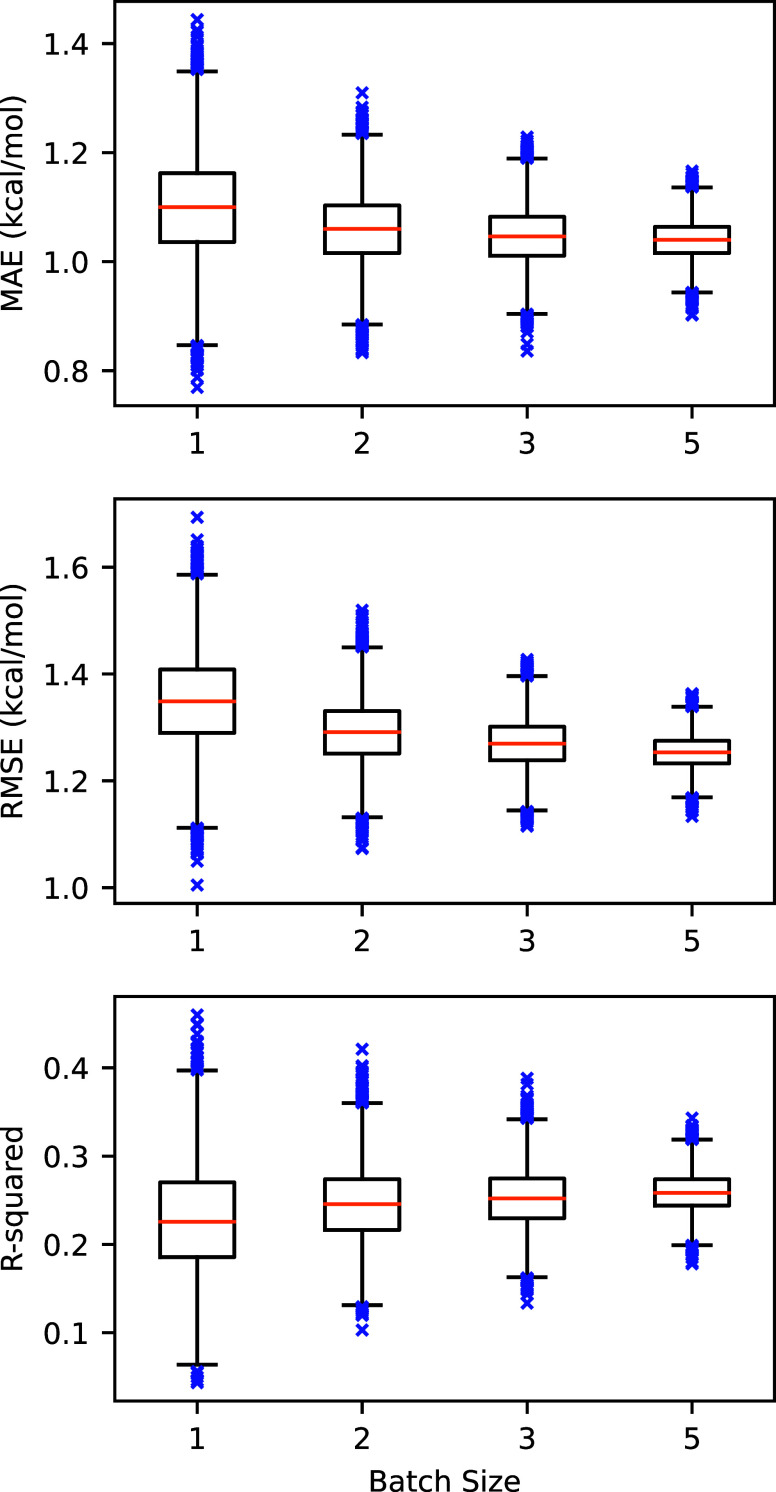
RMSE, MAE, and *R* ^2^ of RBFE calculations for CDK2 employing a nine-point Gaussian quadrature λ-schedule using protocol A with respect to the experimental values. Batch size refers to the number of replicates averaged together per mutation. The box-and-whisker plots were generated using matplotlib with standard parameters. The box represents the interquartile range (IQR) with the median shown in orange. The whiskers represent 1.5 IQR away from the first and third quartiles. Outlier points are shown in blue.

ΔΔ*G*_A→B_^bind^ obtained from protocol C12 and protocols A, B, and C displayed good agreement, with *R* ^2^ values of 0.926, 0.945, and 0.938, respectively, when considering all ten replicates per mutation, as seen in Figure 5.

**Figure 5:**
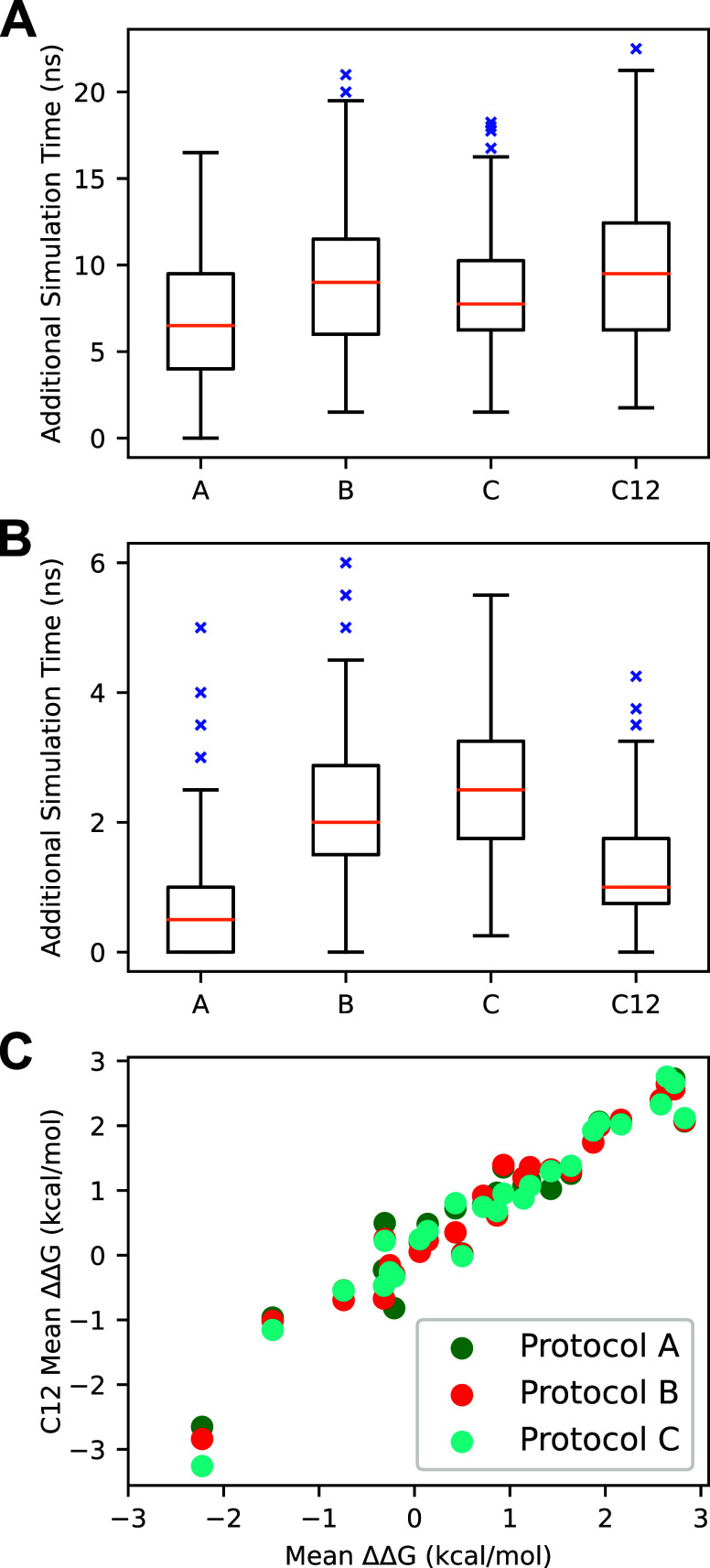
(A) Distributions of protein–ligand complex simulation step additional simulation times for all mutations by protocol. (B) Distributions of solvated ligand simulation step additional simulation times for all mutations by the protocol. (C) Scatterplot of ΔΔ*G*_A→B_^bind^ values from protocol C12 versus protocols (A–C). Protocols A and B utilized 2.5 and 1.5 ns initial simulation lengths, respectively, with a 0.5 ns additional simulation length, while protocol C utilized a 1.0 ns initial simulation length and a 0.25 additional simulation length. Box-and-whisker plots were generated analogously to Figure 4.

The number of additional simulations performed varied by protocol, mutation, λ-window, and alchemical step, as seen in Figure 5. As expected, the protein–ligand complex step required more average additional simulation time to achieve convergence than the solvated ligand step, regardless of the protocol employed. Protocol A generally required the least additional simulation time to achieve convergence in both alchemical steps (see Table 1 for protocol details). Overall, the additional simulation time was evenly distributed between λ-windows of the solvated ligand step, with more variation in the protein–ligand complex step; however, this pattern broke down when comparing specific mutations, as seen in Figure 6. A correlation was observed between the average additional simulation times of the λ-windows of the various protocols, which indicates that our protocol is correctly identifying λ-windows that require additional simulation to achieve convergence.

**Figure 6:**
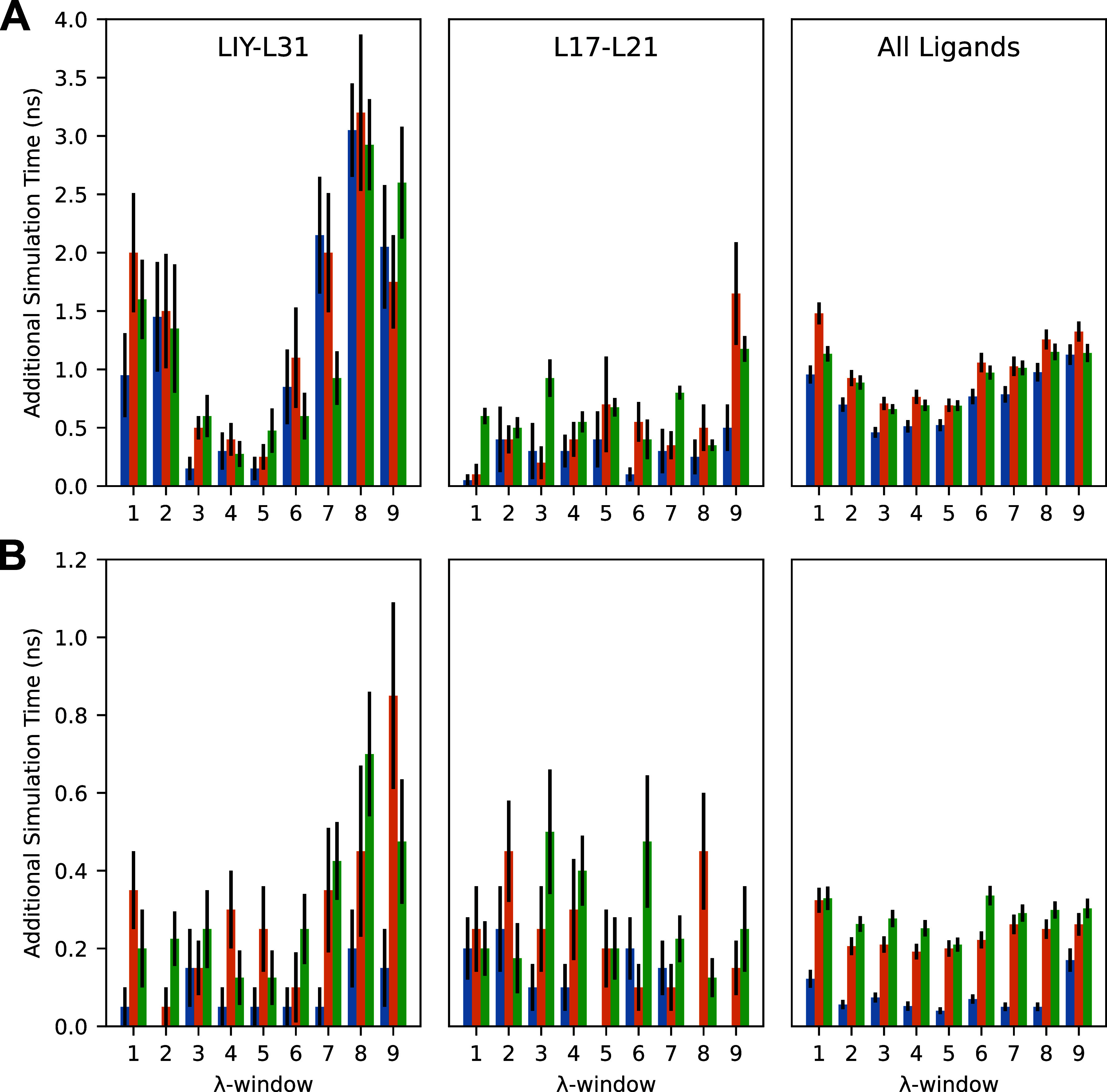
(A) Distributions of average protein–ligand complex step additional simulation time performed per RBFE simulation of the LIY-L31 and L17-L21 mutations and of all 25 mutations. (B) Distributions of average solvated ligand step additional simulation time performed per RBFE simulation of the LIY-L31 and L17-L21 mutations and of all 25 mutations. Protocols A (2.5 ns initial, 0.5 ns additional), B (1.5 ns initial, 0.5 ns additional), and C (1.0 ns initial, 0.25 ns additional) are shown in blue, orange, and green, respectively. Error bars represent one standard error of the mean.

Truncated trajectories of all replicates were examined, with 10,000 MAEs and RMSEs generated via the resampling scheme described above. Mean MAEs and RMSEs and their respective standard errors are displayed in Figure 7. In all protocols, significant accuracy improvement is observed during the first nanosecond of simulation time, with only minor accuracy improvement observed afterward. After the initial simulation period, protocols A and C12 do not display any gain in accuracy, whereas protocols B and C do (see Table 1 for protocol details). These results suggest that a shorter maximum simulation time may be employed without a loss of accuracy.

**Figure 7:**
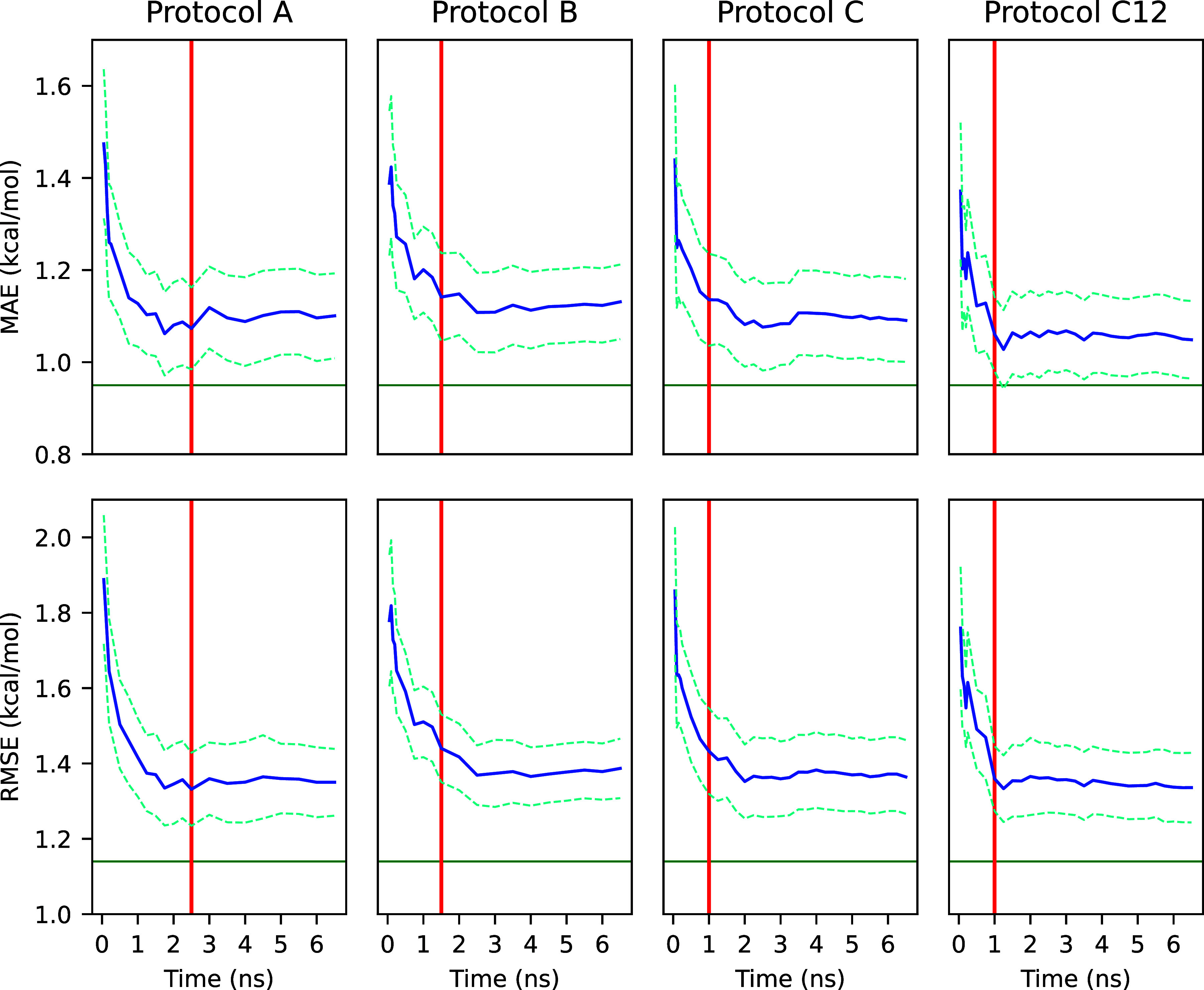
CDK2 MAEs and RMSEs calculated from truncated gradients. The *x*-axis shows the truncation time of all trajectories used to compute the corresponding *y*-value and uncertainty. Note that not all trajectories were necessarily extended through the entire domain of the *x*-axis. The blue line represents the respective loss function (MAE or RMSE), the dashed line represents one standard error of the loss function, the red line represents the length of the initial simulation period for all simulations of a given protocol, and the green line represents the value of the respective loss function achieved by Song et al. Protocols A and B utilized 2.5 and 1.5 ns initial simulation lengths, respectively, with a 0.5 ns additional simulation length, while protocol C utilized a 1.0 ns initial simulation length and a 0.25 ns additional simulation length. Protocol C12 utilizes a 1.0 ns initial simulation and 0.25 ns additional simulations, a 1 fs time step (as opposed to 2 fs), 12 λ-windows (as opposed to 9), a Berendsen thermostat (as opposed to a Langevin thermostat) and does not utilize the SHAKE algorithm, unlike the other protocols.

Computational cost savings for both the protein–ligand complex and solvated ligand steps were calculated by taking 10,000 independent samples of a particular batch size of each mutation and summing up the total number of production nanoseconds of a batch of a given mutation for a given alchemical step. For protocols A, B, and C, the complement of this number divided by 120 was taken, representing the total number of nanoseconds simulated by Song et al. for a given mutation multiplied by two to account for their use of 1 fs time step. For protocol C12, the complement of the sum simulation time divided by 60 was used. Means and standard errors of savings were calculated from these distributions. The summary statistics of these savings are given in Table S1.

Overall, the accuracy of the optimized RBFE calculations was comparable to the benchmark results, regardless of the protocol employed. The most directly comparable result—a single simulation per mutation using protocol C12—had an MAE of 1.05 ± 0.08, with comparable results achieved with protocols A, B, and C (see Table S1). These values are on average slightly greater than the MAE and RMSE of 0.95 and 1.14 kcal/mol, respectively, reported by Song et al., but the difference in MAE is statistically insignificant for protocols A, C, and C12 at the 95% confidence level. However, these results were achieved with more than a 64% reduction in production computational cost. In the case of protocol C, the average savings exceeded 85%. All protocols and all batch sizes achieved *R* ^2^ values greater than the 0.15 reported by Song et al. (see Table S1). Repeated runs, on average, appear to have little benefit, as the average MAE and RMSE decrease only moderately, and the average *R* ^2^ increases only slightly when increasing the batch size from one to ten replicates for all protocols. However, one can see in Figure 4 that the spread of the MAE, RMSE, and *R* ^2^ distributions significantly decreases with increasing batch size, meaning that the likelihood of calculating a set of particularly poor (or outstanding) RBFEs decreases with repeated runs. This tradeoff should be considered when planning a high-throughput RBFE campaign: if one can accept a higher variance of calculations, then significantly more mutations can be explored at a similar cost.

### RBFE Calculations for PLpro

For each mutation, three 10–15 ns production simulations were performed at each λ-window (see Figure S1 for details on the extension of the initial 10 ns trajectory). The gradients were extracted and analyzed with three different equilibration methods: AED (see Approach), a 2 ns equilibration period, and a 5 ns equilibration period. The equilibrated gradients were then decorrelated, and averages were extracted. Ten short-run simulations were performed with protocols A, B, C, and D for each mutation (see Table 1 for protocol details).

As with the CDK2 mutations, the amount of simulation time applied to each λ-window varied by λ-window, simulation protocol, alchemical step, and mutation, as seen in Figure 8. We note that λ-windows of the protein–ligand complex step with elevated simulation times in protocol A also tended to have elevated simulation times in protocols B–D, and the same pattern holds with respect to protocols B and C. This demonstrates that different λ-windows converge at different rates and that this pattern is consistent. This pattern is not clear in the solvated ligand step as these λ-windows converged much faster than the protein–ligand complex step.

**Figure 8:**
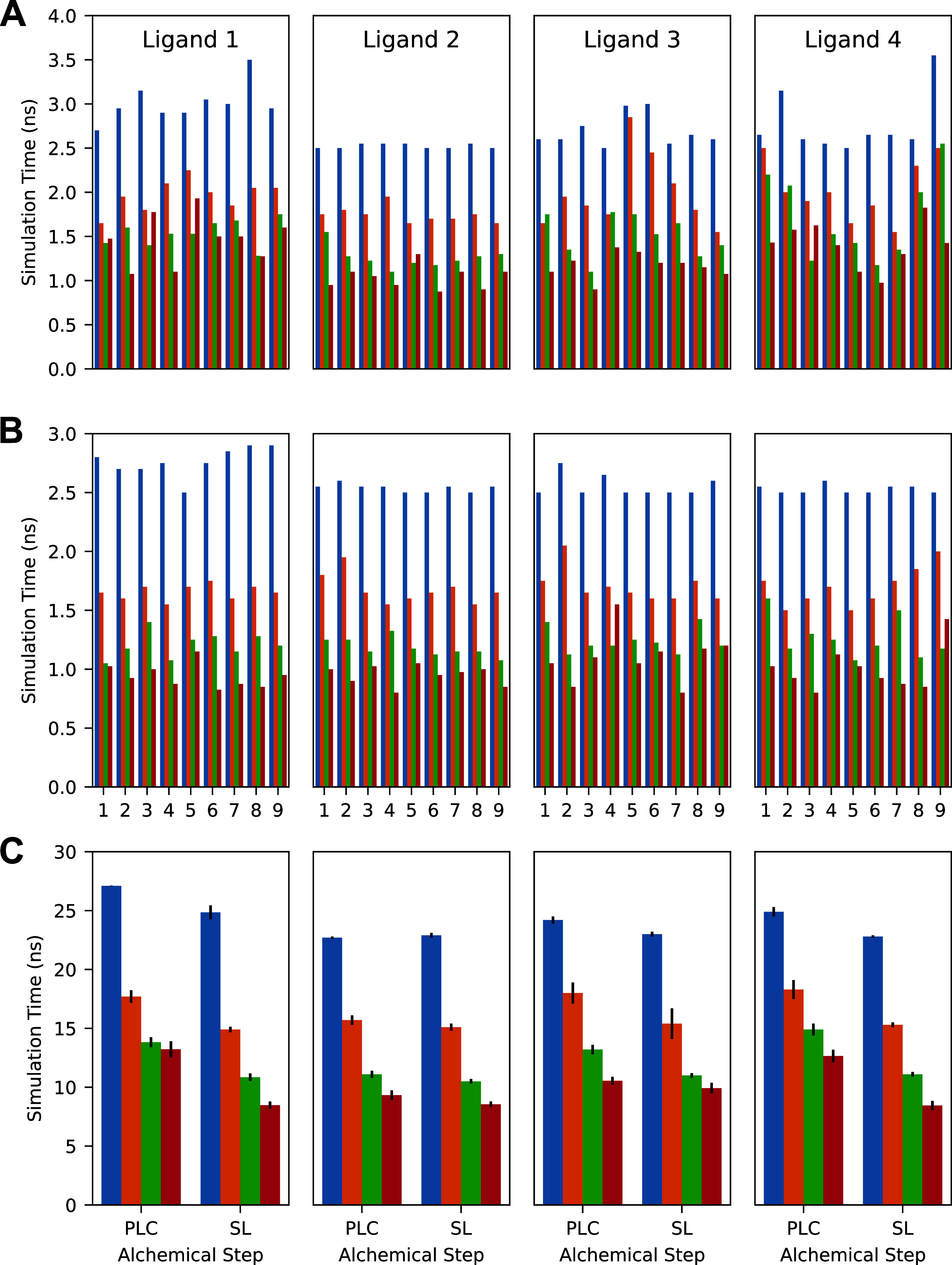
(A) Average simulation time applied to each λ-window of the protein–ligand complex step by protocol and mutation. (B) Average simulation time applied to the solvated ligand step by protocol and mutation. (C) Overall average applied simulation time per replicate by mutation, alchemical step, and protocol. Blue bars represent protocol A (2.5 ns initial, 0.5 ns additional), orange bars represent protocol B (1.0 ns initial, 0.5 ns additional), green bars represent protocol C (1.0 ns initial, 0.25 ns additional), and red bars represent protocol D (0.5 ns initial, 0.25 ns additional). Error bars denote one standard error.

**Figure 9:**
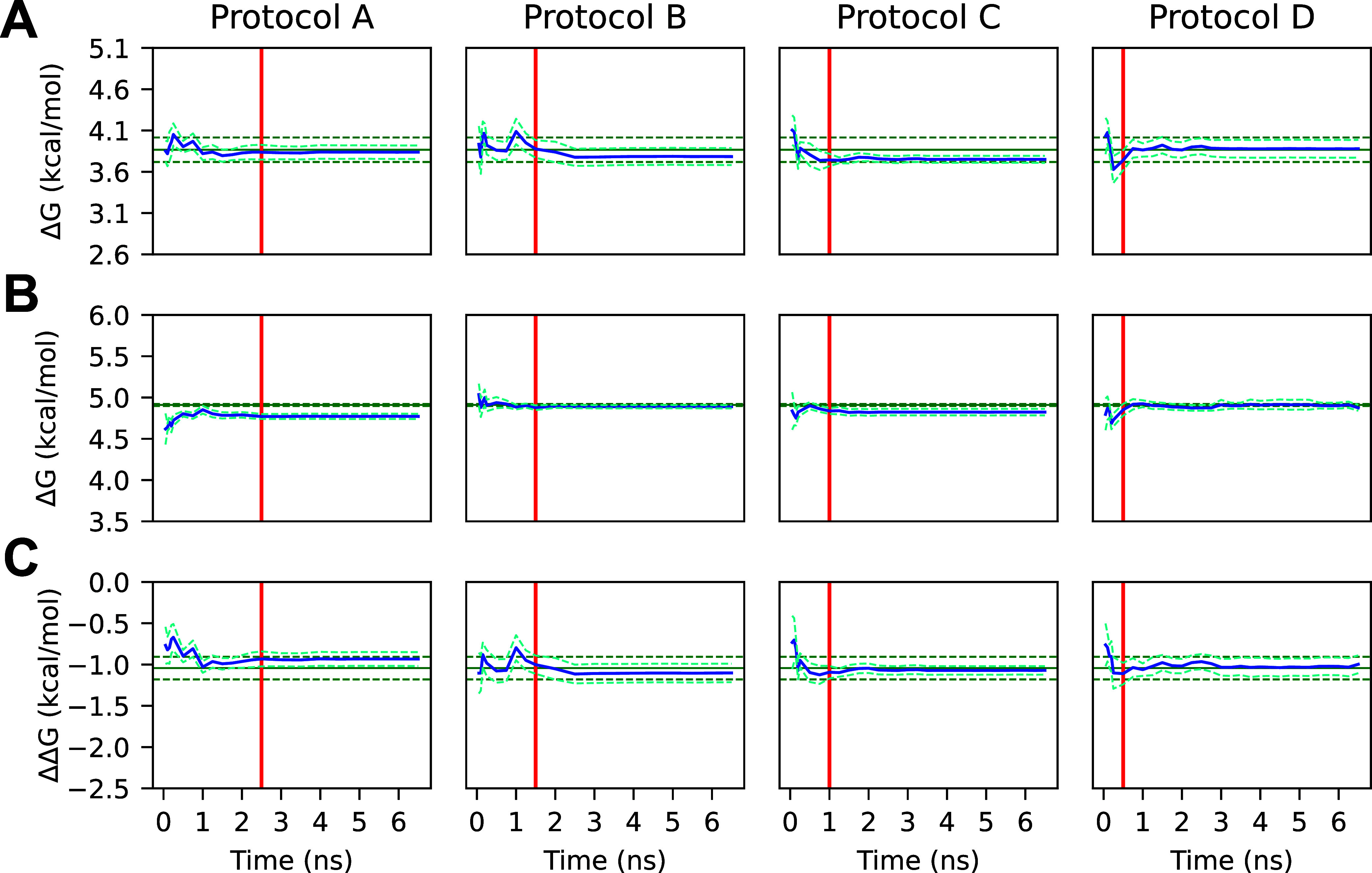
(A) Average ligand 1 protein–ligand complex ΔG calculated from truncated gradients. (B) Average ligand 1 solvated ligand ΔG calculated from truncated gradients. (C) Average ΔΔ*G*_ref→1_^bind^ calculated from truncated trajectories. The *x*-axis shows the truncation time of all trajectories used to compute the corresponding *y*-value and uncertainty. Note that not all trajectories were necessarily extended through the entire domain of the *x*-axis. The blue line represents the mean Δ*G* or ΔΔ*G*_ref→1_^bind^, and the blue dashed line represents one standard error of the mean. The green line represents the mean long-run Δ*G* or ΔΔ*G*_ref→1_^bind^ calculated using AED, the green dashed line represents one standard error of the mean, and the red line denotes the length of the initial simulation for all simulations of the given protocol. Protocols A and B utilized 2.5 and 1.5 ns initial simulation lengths, respectively, with a 0.5 ns additional simulation length. Protocols C and D utilized 1.0 and 0.5 ns initial simulation lengths, respectively, with a 0.25 ns additional simulation length. Convergence with the long-run simulations was typically achieved quickly, with a similar pattern observed for ligands 2 and 3 (see Figures S2 and S3).

The average protein–ligand complex Δ*G*, solvated ligand Δ*G*, and overall ΔΔ*G*_A→B_^bind^ of the short-run and long-run simulations with all three equilibration methods are tabulated in Table 2. ΔΔ*G*_A→B_^bind^ and alchemical step Δ*G* values obtained by each short-run protocol were within 1.1 kcal/mol to those obtained by the long-run protocols. ΔΔ*G*_ref→4_^bind^ values deviated more from their long-run counterparts than those obtained for ligands 1–3, with an absolute error of 0.6–1.1 kcal/mol compared to 0.1–0.2 kcal/mol, respectively. For ligand 4, the protein–ligand complex step was the major contributor to the absolute error (0.6–1.0 kcal/mol), while the contribution of the solvated ligand step was considerably smaller (0.1–0.3 kcal/mol). This deviation did not alleviate with more simulation time in protocols A–D (Figure 10), indicating that the relevant time scales are outside the length of these short simulations (see Table 1 for protocol details). The larger error obtained for ligand 4 can be explained by the difficulty of the mutation, which involves the mutation of the amine group into the methylaminosulfonyl group, a benzene ring methyl group into chlorine, and a naphthalene ring hydrogen into chlorine. This involves the mutation of seven heavy atoms and the overall addition of two rotatable bonds (Figure 11). The methylaminosulfonyl group, which is flexible and solvent-exposed, covers a larger area of the phase space and is thus more difficult to converge and requires more simulation time, as seen in Figure 12. Due to the presence of two rotatable bonds (C–S and S–N), the conformation of methylaminosulfonyl group can vary significantly during RBFE simulations (see Figures 11D,E, S5, and S6). These conformations differ by interactions with the closest protein residues (D164, E167, Y268, and Q269; see Figure 11B,C). This results in considerable fluctuations in $\frac{dV}{d\lambda }$ gradient time series and therefore in a slow convergency of $\left\langle \frac{dV}{d\lambda }\right\rangle$ at some λ-windows (see Figure S6).

**Figure 10:**
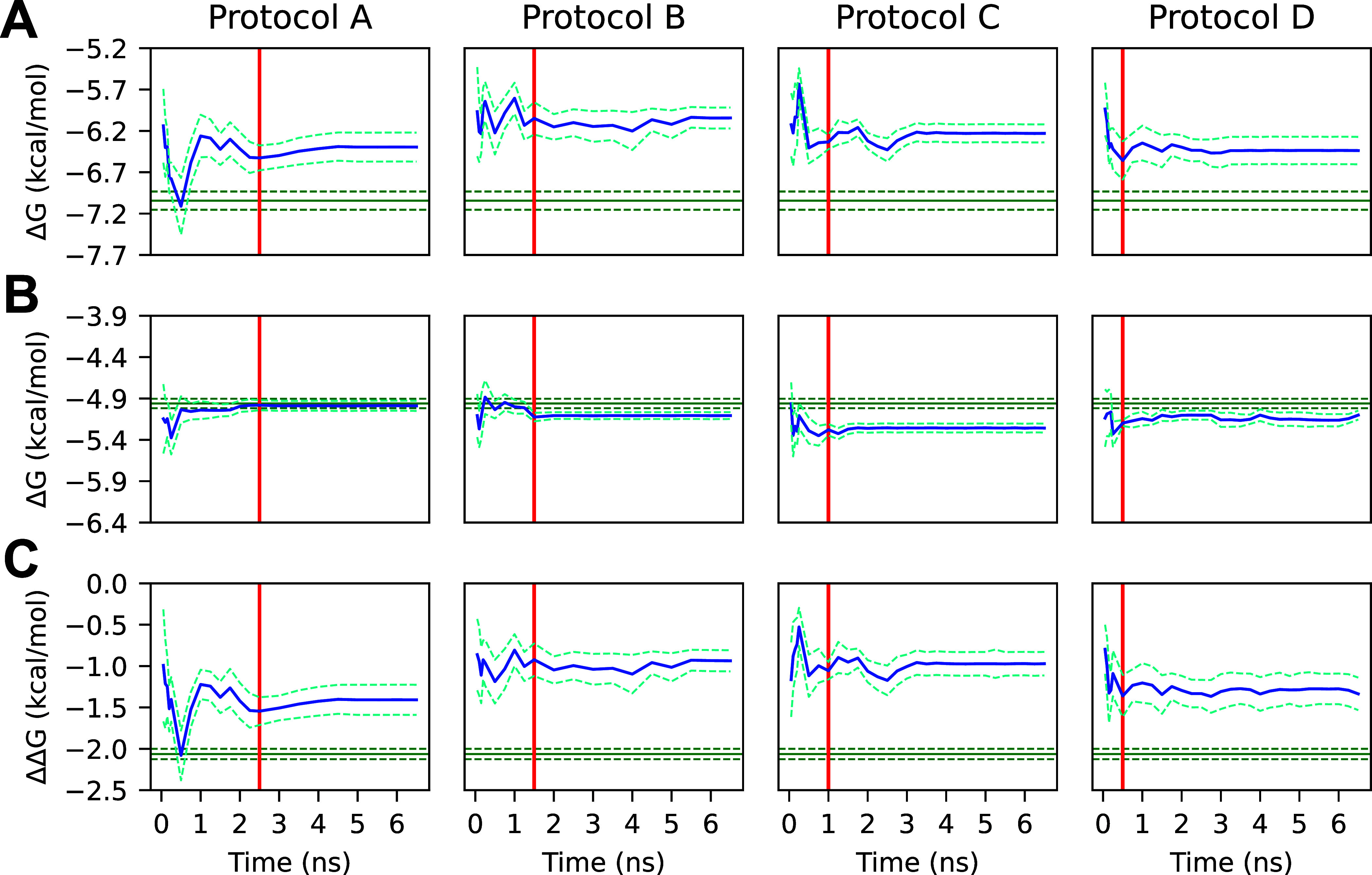
(A) Average ligand 4 protein–ligand complex Δ*G*s calculated from truncated gradients. (B) Average ligand 4 solvated ligand Δ*G*s calculated from truncated gradients. (C) Average ΔΔ*G*_ref→4_^bind^ calculated from truncated trajectories. The *x*-axis shows the truncation time of all trajectories used to compute the corresponding *y*-value and uncertainty. Note that not all trajectories were necessarily extended through the entire domain of the *x*-axis. The blue line represents the mean Δ*G* or ΔΔ*G*_ref→4_^bind^, and the blue dashed line represents one standard error of the mean. The green line represents the mean long-run Δ*G* or ΔΔ*G*_ref→4_^bind^ calculated using AED, the green dashed line represents one standard error of the mean, and the red line denotes the length of the initial simulation for all simulations of the given protocol. Protocols A and B utilized 2.5 and 1.5 ns initial simulation lengths, respectively, with 0.5 ns additional simulation lengths. Protocols C and D utilized 1.0 and 0.5 ns initial simulation lengths, respectively, with a 0.25 ns additional simulation length. A significant deviation in the protein–ligand complex step for this difficult mutation is observed between all short-run and long-run protocols.

**Figure 11:**
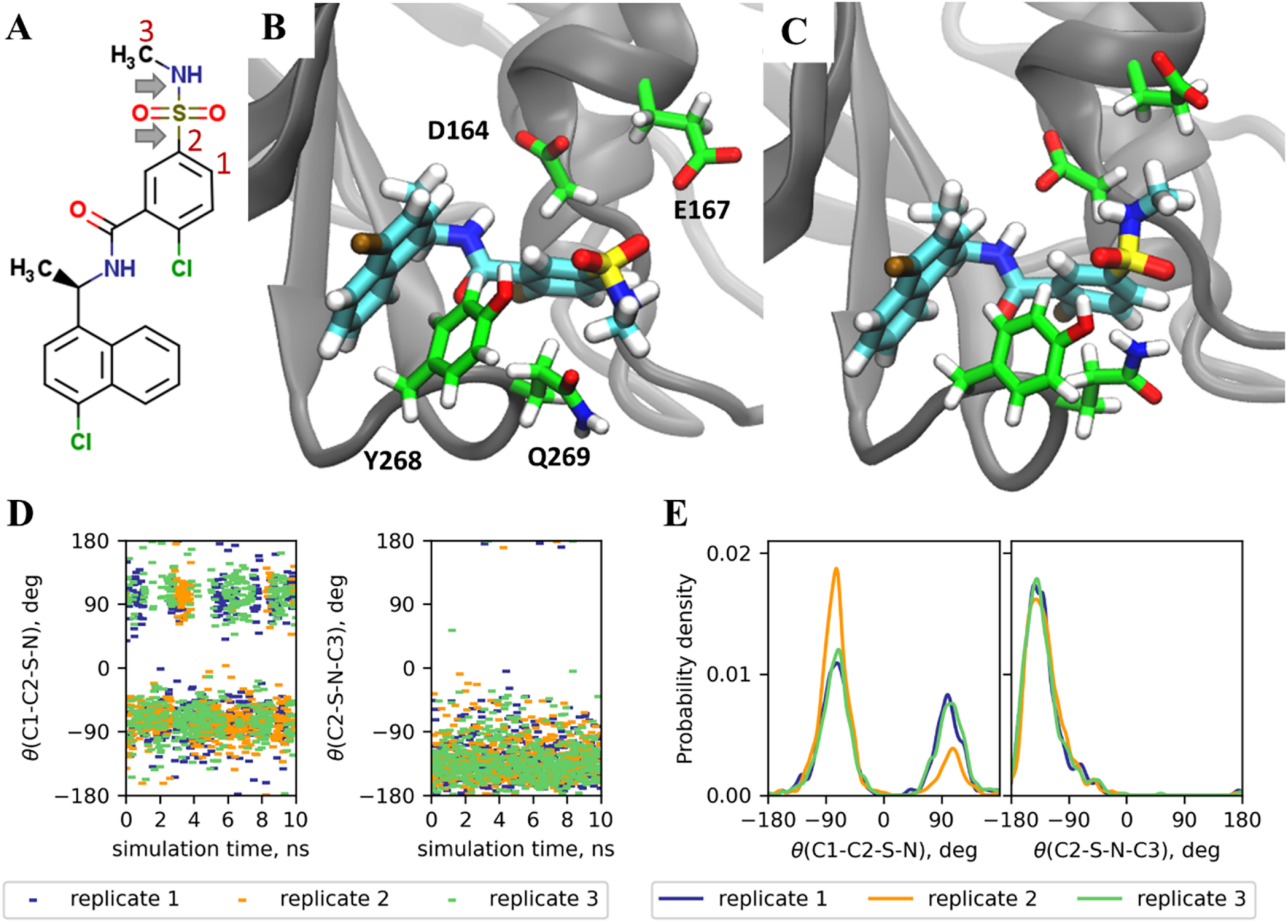
Fluctuations of the methylaminosulfonyl group of ligand 4 in the protein–ligand complex step of RBFE simulations for PLpro. (A) Structure of ligand 4. Rotatable bonds of the methylaminosulfonyl group are indicated by gray arrows. Carbon atoms forming the corresponding dihedral angles are numbered. (B, C) Structures of PLpro in complex with ligand 4 for the ligand conformation with C1–C2–S–N dihedral angles of approximately 90° (B) and −90° (C) extracted from long-run MD simulations at λ = 0.5. The protein backbone is shown as the gray cartoon, and the ligand and side chains of residues within 5 Å of the methylaminosulfonyl group are shown as sticks. (D) Fluctuation of dihedral angles C1–C2–S–N (left) and C2–S–N–C3 (right) during long-run MD simulations at λ = 0.5. The three independent replicates of long-run MD simulations are shown in green, orange, and blue. (E) Distribution of dihedral angles C1–C2–S–N (left) and C2–S–N–C3 (right) at long-run MD simulations at λ = 0.5.

**Figure 12:**
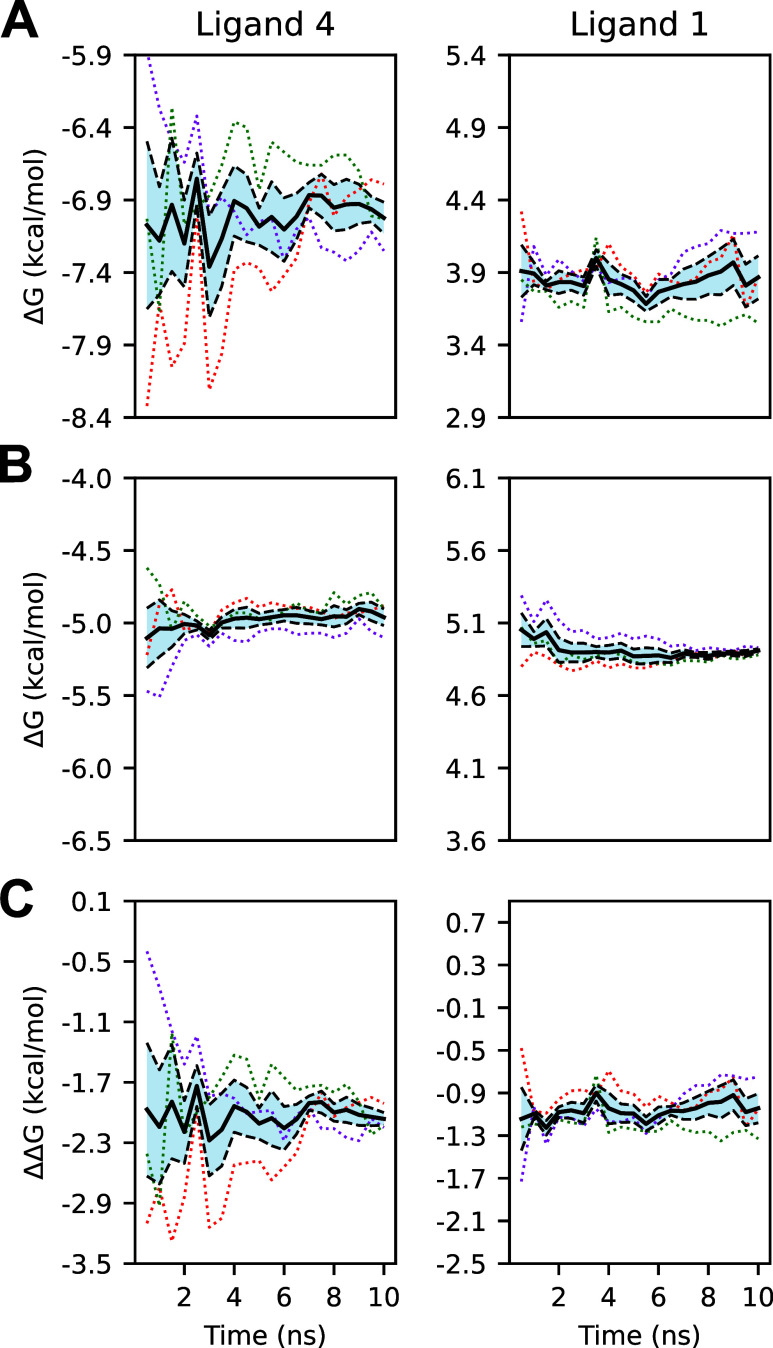
(A) Protein–ligand complex ΔGs from truncated gradients of long-run simulations calculated with AED. (B) Solvated ligand ΔGs from truncated gradients of long-run simulations calculated with AED. (C) ΔΔ*G*_A→B_^bind^ values from truncated gradients of long-run simulations calculated with AED. The *x*-axis shows the truncation time of all trajectories used to compute the corresponding *y*-value and uncertainty. Dotted lines represent individual replicates, while the black solid and dashed lines represent means and standard errors, respectively. Ligand 4 does not converge until approximately 4 ns of simulation time, whereas ligand 1 converges much more quickly.

**Table 2:** Average RBFEs and Their Components of Short-Run and Long-Run Simulations for PLpro by the Alchemical Step, Ligand (L), Equilibration Method (eq.), and Protocol

		long run (kcal/mol)			short run (kcal/mol)			
Alchemical step	L	AED eq.	2 ns eq.	5 ns eq.	Protocol A	Protocol B	Protocol C	Protocol D
protein–ligand complex	1	3.87 ± 0.15	3.80 ± 0.11	3.80 ± 0.16	3.84 ± 0.08	3.78 ± 0.10	3.75 ± 0.04	3.88 ± 0.10
	2	–1.96 ± 0.03	–1.98 ± 0.03	–1.99 ± 0.04	–1.93 ± 0.02	–1.90 ± 0.03	–1.98 ± 0.05	–1.79 ± 0.03
	3	4.75 ± 0.11	4.82 ± 0.09	4.82 ± 0.14	4.80 ± 0.04	4.83 ± 0.04	4.75 ± 0.04	4.90 ± 0.07
	4	–7.02 ± 0.13	–7.03 ± 0.22	–6.97 ± 0.28	–6.40 ± 0.18	–6.04 ± 0.13	–6.23 ± 0.12	–6.44 ± 0.17
solvated ligand	1	4.91 ± 0.01	4.84 ± 0.02	4.88 ± 0.04	4.77 ± 0.03	4.89 ± 0.02	4.82 ± 0.04	4.87 ± 0.03
	2	–2.06 ± 0.01	–2.07 ± 0.01	–2.06 ± 0.05	–2.09 ± 0.02	–2.10 ± 0.00	–2.12 ± 0.02	–1.98 ± 0.03
	3	3.50 ± 0.02	3.50 ± 0.02	3.53 ± 0.01	3.52 ± 0.02	3.54 ± 0.00	3.51 ± 0.02	3.53 ± 0.03
	4	–4.96 ± 0.07	–4.94 ± 0.06	–4.90 ± 0.10	–4.99 ± 0.06	–5.11 ± 0.01	–5.26 ± 0.06	–5.10 ± 0.05
Total RBFE (ΔΔ*G*_A→B_^bind^)	***1***	**-1.04 ± 0.14**	**-1.04 ± 0.09**	**-1.09 ± 0.13**	**-0.93 ± 0.08**	**-1.10 ± 0.11**	**-1.07 ± 0.05**	**-1.00 ± 0.11**
	***2***	**0.10 ± 0.02**	**0.09 ± 0.03**	**0.09 ± 0.05**	**0.16 ± 0.03**	**0.20 ± 0.04**	**0.14 ± 0.05**	**0.19 ± 0.06**
	***3***	**1.24 ± 0.13**	**1.32 ± 0.07**	**1.29 ± 0.13**	**1.28 ± 0.04**	**1.28 ± 0.05**	**1.24 ± 0.04**	**1.36 ± 0.07**
	***4***	**-2.06 ± 0.08**	**-2.09 ± 0.17**	**-2.06 ± 0.25**	**-1.41 ± 0.19**	**-0.94 ± 0.13**	**-0.97 ± 0.15**	**-1.34 ± 0.20**

To evaluate this discrepancy, we tested protocol E on ligand 4 (see Table 1 for protocol details), which yielded mean values of −6.61 ± 0.12, −5.01 ± 0.04, and −1.60 ± 0.12 kcal/mol for the protein–ligand complex step, the solvated ligand step, and the overall calculation, respectively. Protocol E therefore provided the smallest absolute error in ΔΔ*G*_ref→4_^bind^ with respect to long runs (0.46–0.49 kcal/mol) compared to other protocols. This suggests that for difficult mutations, longer initial simulation times are necessary.

For the protein–ligand complex step of ligand 4, protocols A and D obtained the smallest deviation (∼0.6 kcal/mol) regardless of equilibration protocol (see Table 1 for protocol details). For ligands 1–3, all protocols performed similarly. As one can see from Figure 9, convergence with the long-run simulations was achieved quickly, which supports the use of short protocols for these smaller RBFE mutations. Overall, protocol B utilized approximately 75 and 65% of the computational resources utilized by protocol A for the protein–ligand complex step and solvated ligand step, respectively, whereas protocols C and D both utilized approximately 50 and 45%, respectively. This was achieved with no accuracy penalty for ligands 1–3 and a moderate accuracy penalty for ligand 4 (∼0.47, ∼0.43, and ∼0.07 kcal/mol, respectively).

### ABFE Calculations for Lysozyme

Four different simulation protocols were utilized: protocols A, B, C (see Table 1 for protocol details), and O, which was utilized in our previous high-throughput screening study^7^ and was performed as a control protocol to evaluate the performance of our other protocol implementations. Protocol O consisted of 4.5 ns of production simulation time per λ-window. The gradient time series were then extracted, equilibrated with a 0.5 ns equilibration period, and decorrelated. For each protocol, the average Δ*G*_bind_^o^ was computed as an average of 39 independent calculations. The experimental Δ*G*_bind_^o^ value of −5.52^5^ kcal/mol was used to evaluate the protocol performance. The average Δ*G*_bind_^o^ values computed with protocols A, B, C, and O were −5.31 ± 0.12, −5.59 ± 0.14, −5.46 ± 0.19 kcal/mol, and −5.36 ± 0.14 kcal/mol, respectively. The MAEs and RMSEs of all four protocols are listed in Table 3. Note that values of batch sizes greater than one were calculated by resampling every combination of Δ*G*_bind_^o^ values of a given batch size and then averaging each sample.

**Table 3:**
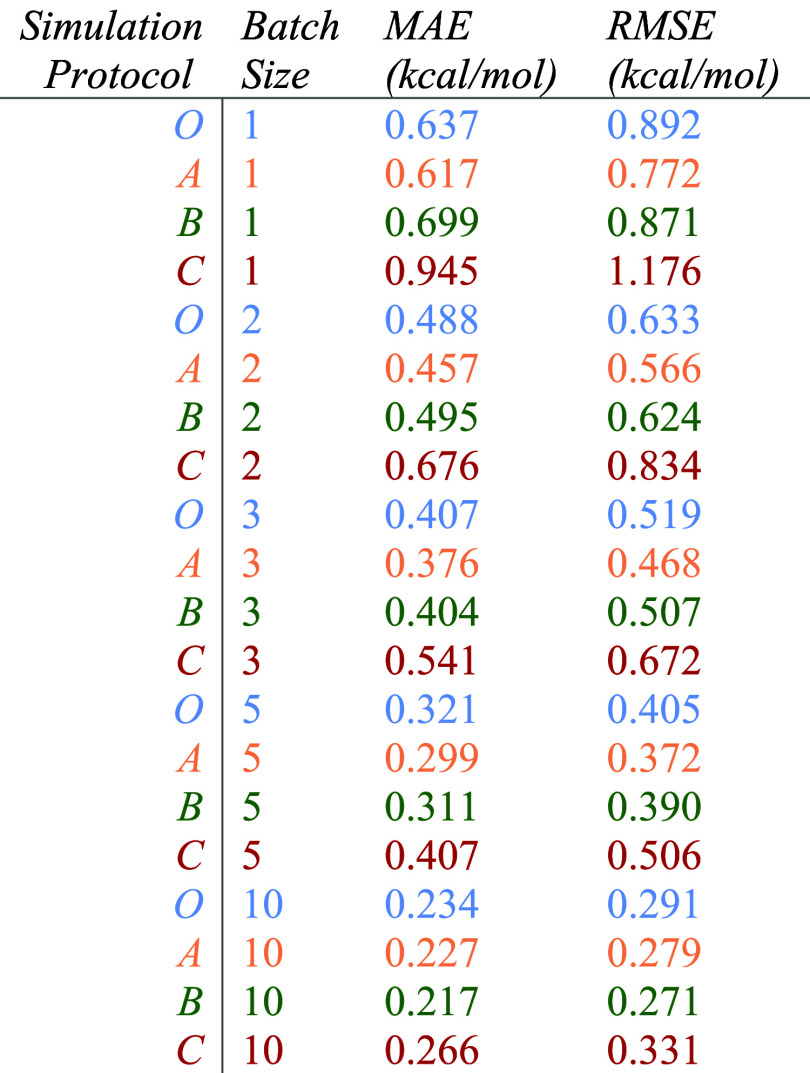
MAEs and RMSEs of 39 Independent ABFE Simulations of Lysozyme

In general, the MAE and RMSE of the control group (protocol O) were comparable to those of protocols A and B across all batch sizes, whereas protocol C was significantly less accurate. Interestingly, protocol A outperformed protocol O with all batch sizes in both MAE and RMSE. Protocol B managed the same feat with batch sizes of 3, 5, and 10 in MAE and all batch sizes in RMSE. Significant computational savings were achieved on all alchemical steps using either protocol A, B, or C, with protocol C achieving approximately 60% average savings or greater across all alchemical steps (see Figure S7), albeit with a significant accuracy penalty. Protocol A was able to improve accuracy across the board when measured by RMSE while achieving average savings of approximately 30–45% depending on the alchemical step. Of the protocols tested, protocol B offered the best compromise between cost and accuracy with a batch size of 1, with approximately a 50% reduction in computational cost with comparable accuracy to protocol O. All protocols and batch sizes obtained an average error within 1 kcal/mol, which is comparable to the corresponding error reported in other studies.^29,81,82^

As seen in Figure 13, the most significant accuracy gains were observed during the first nanosecond of production simulation time for protocols A and B, whereas protocol C displayed more muted gains. Protocol C displayed elevated inaccuracy at 1 ns of production simulation time compared to protocols A and B at the same time period, despite all protocols being equivalent at this point (see Table 1 for protocol details). This may indicate that protocol C suffered from an uncommonly inaccurate batch of simulations, which may help explain its relative underperformance. All protocol average Δ*G*_bind_^o^ converge within 1–2 ns to within a standard error of the experimental value, which indicates that shorter maximum simulation times may be employed while maintaining accuracy.

**Figure 13:**
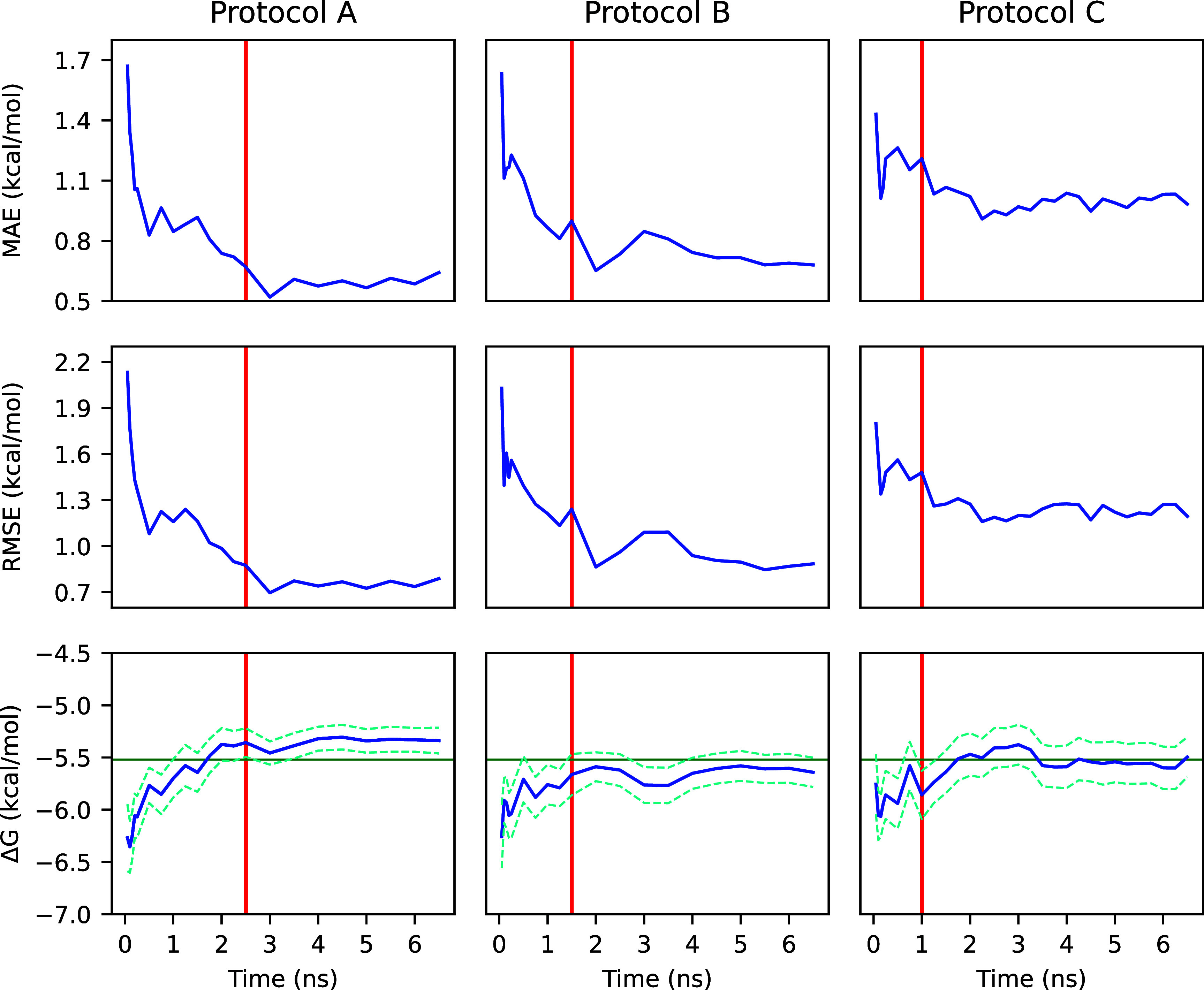
Lysozyme MAE, RMSE, and overall Δ*G*_bind_^o^ values calculated from truncated gradients of 39 independent simulations. The *x*-axis shows the truncation time of all trajectories used to compute the corresponding *y*-value and uncertainty. Note that not all trajectories were necessarily extended through the entire domain of the *x*-axis. Protocols A and B utilized 2.5 and 1.5 ns initial simulation lengths, respectively, with a 0.5 ns additional simulation length, while protocol C utilized a 1.0 ns initial simulation length and a 0.25 ns additional simulation length. The dashed line represents one standard error of the mean value, the red line represents the length of the initial simulation period for all simulations of the given protocol, and the green line represents the experimental value of Δ*G*_bind_^o^.

As opposed to the RBFE results, repeated runs had a significant impact on MAE and RMSE. Within protocol O, MAE decreased from 0.637 to 0.234 kcal/mol when moving from a batch size of one to ten. Protocols A, B, and C showed similar trends, with MAE decreasing from 0.617 to 0.227 kcal/mol, from 0.699 to 0.217 kcal/mol, and from 0.945 to 0.266, respectively. The difference in MAE between protocols A and B decreased from 0.082 to 0.010 kcal/mol, with protocol B becoming superior as the batch size increased from 1 to 10. Similar results were achieved when comparing RMSE values, with protocol B displaying an approximately threefold RMSE decrease and the difference between protocols A and B RMSEs decreasing from 0.101 to 0.008 kcal/mol, with protocol B becoming superior as the batch size increased from one to ten replicates. These results suggest that in high-throughput virtual screening campaigns utilizing ensembles of ABFE simulations, protocol A or B will outperform other protocols with uniform resource allocation. Furthermore, while protocol A can achieve higher accuracy in one-off simulations, albeit with significantly higher cost, this advantage evaporates as batch size increases. At a batch size of ten replicates, significant savings are realized with protocol B with no relative accuracy penalty.

### ABFE PLpro

Three 100 ns simulations were performed at each λ-window. The gradients of the short-run simulations were evaluated against those of the long-run simulations in an analogous manner, as described for the RBFE simulations. Similarly to the RBFE PLpro study, the amount of simulation time applied to each λ-window varied by the λ-window, simulation protocol, and alchemical step (see Figure S8). We note once again that λ-windows in the protein–ligand complex and restraint addition alchemical steps with elevated simulation times in protocol A tended to have elevated simulation times in protocols B and C, with an analogous pattern holding for protocols B and C (see Table 1 for protocol details).

For all protocols, the protein–ligand complex and solvated ligand step short-run simulations, as well as the overall Δ*G*_bind_^o^ short-run simulations, converged quickly toward their respective long-run averages and were within error after 1–2 ns of simulation time, as seen in Figure 14. The restraint addition step short-run simulations, however, remained well outside of error. Analysis of this alchemical step showed that regardless of the equilibration protocol employed, over 40 ns of production simulation time per λ-window is required to achieve the average values of approximately 2 kcal/mol (Figure 15), which is significantly more resources than can be dedicated for this purpose.

**Figure 14:**
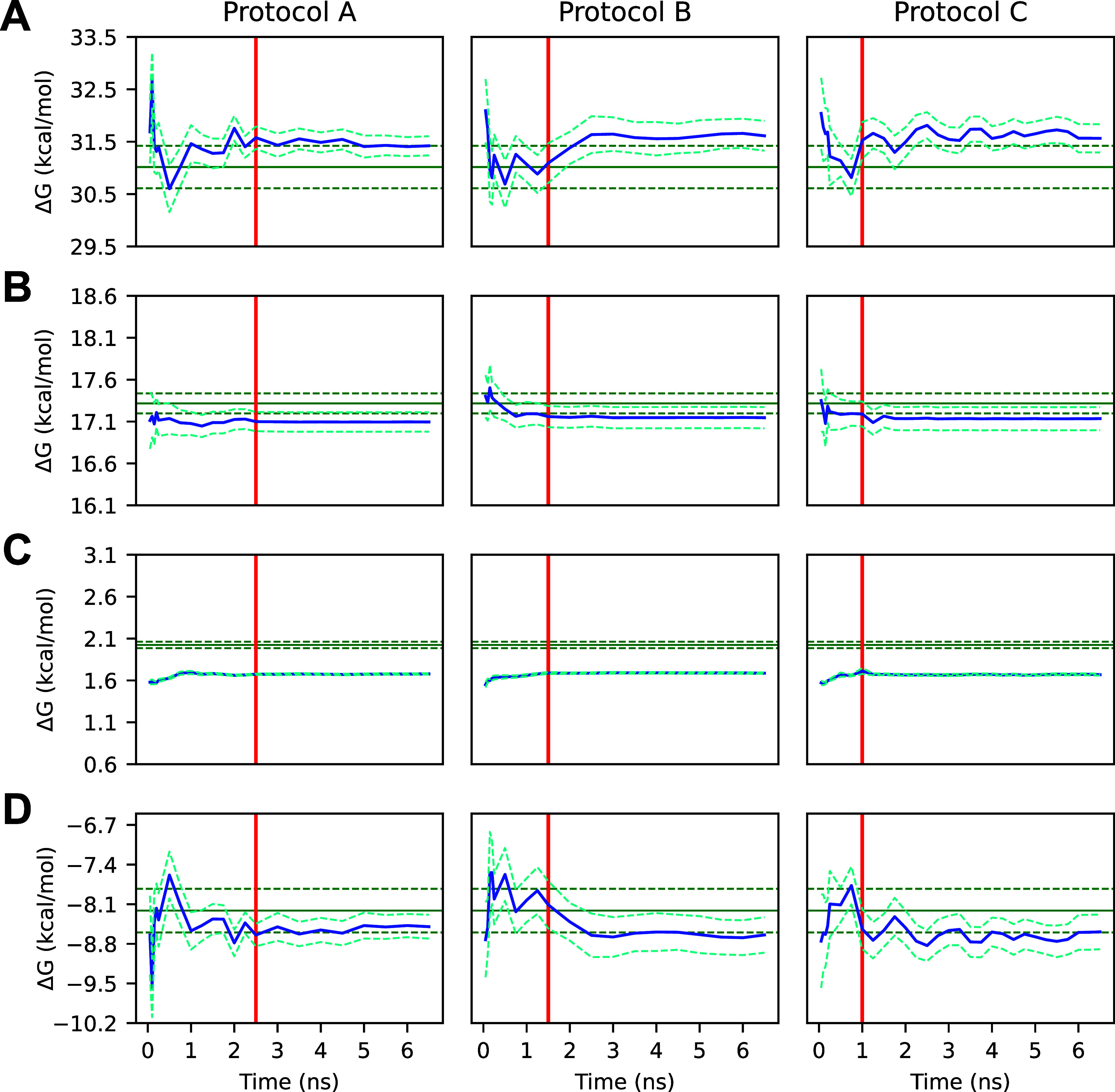
(A) PLpro ABFE protein–ligand complex step ΔGs calculated from truncated gradients by protocol. (B) PLpro ABFE solvated ligand step ΔGs calculated from truncated gradients by protocol. (C) PLpro ABFE restraint addition step ΔGs calculated from truncated gradients by protocol. (D) PLpro ABFE overall Δ*G*_bind_^o^ values calculated from truncated gradients by protocol. The *x*-axis shows the truncation time of all trajectories used to compute the corresponding *y*-value and uncertainty. Note that not all trajectories were necessarily extended through the entire domain of the *x*-axis. Protocols A and B utilized 2.5 and 1.5 ns initial simulation lengths, respectively, with a 0.5 ns additional simulation length, while protocol C utilized a 1.0 ns initial simulation length and a 0.25 ns additional simulation length. The blue solid line represents the mean short-run value, the blue dashed line represents one standard error of the mean short-run value, the green solid line represents the long-run mean value calculated with AED, the green dashed line represents one standard error of the long-run mean value, and the red line represents the length of the initial simulation period for all simulations of the given protocol.

**Figure 15:**
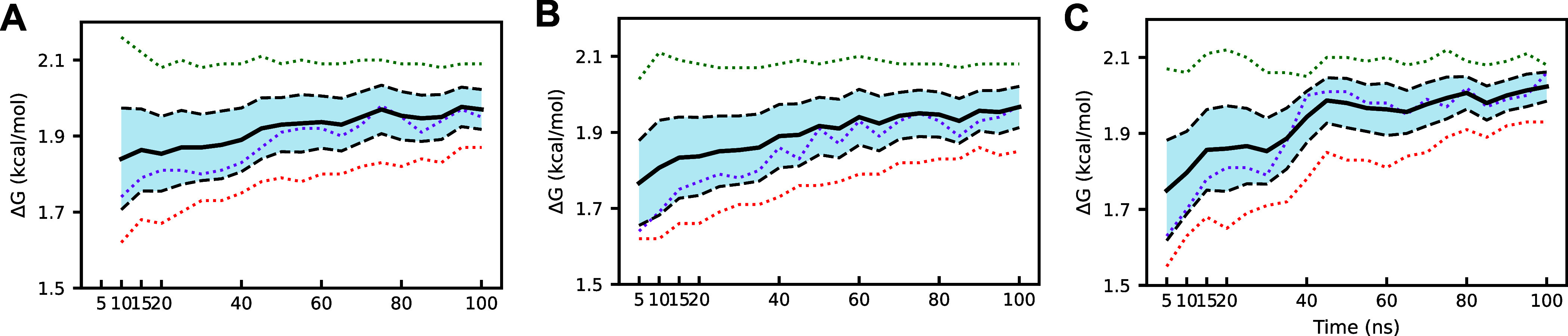
(A) PLpro ABFE long-run restrain addition step ΔG calculated from truncated gradients with a 5 ns equilibration period. (B) PLpro ABFE long-run restrain addition step Δ*G* calculated from truncated gradients with a 2 ns equilibration period. (C) PLpro ABFE long-run restrain addition step Δ*G* calculated from truncated gradients with AED. The dotted lines each represent an individual replicate, while the black solid and dashed lines represent the average value and standard error, respectively. We note that the overall uncertainty was minimized with the AED equilibration protocol.

The average protein–ligand complex Δ*G*, solvated ligand Δ*G*, restraint addition Δ*G*, and overall Δ*G*_bind_^o^ values of the short-run and long-run simulations with all three equilibration methods were computed and are tabulated in Table 4.

**Table 4:** Δ*G*s of Short-Run and Long-Run PLpro ABFE Simulations by the Alchemical Step

	overall ΔG_bind_^o^ (kcal/mol)	protein–ligand complex (kcal/mol)	solvated ligand (kcal/mol)	restraint addition (kcal/mol)	protein–ligand complex simulation time (ns)	solvated ligand simulation time (ns)	restraint addition simulation time (ns)
long-run AED equilibration	–8.21 ± 0.47	31.02 ± 0.50	17.32 ± 0.15	2.02 ± 0.05	900	900	700
long-run 2 ns equilibration	–7.65 ± 0.85	30.50 ± 0.91	17.33 ± 0.15	1.97 ± 0.07	900	900	700
long-run 5 ns equilibration	–7.85 ± 0.67	30.71 ± 0.71	17.32 ± 0.16	1.97 ± 0.06	900	900	700
Protocol A	–8.50 ± 0.21	31.42 ± 0.18	17.10 ± 0.12	1.68 ± 0.01	27.8 ± 0.4	22.9 ± 0.1	26.5 ± 0.4
Protocol B	–8.64 ± 0.31	31.61 ± 0.28	17.15 ± 0.13	1.69 ± 0.01	20.1 ± 0.6	15.7 ± 0.2	19.2 ± 0.5
Protocol C	–8.59 ± 0.31	31.56 ± 0.27	17.14 ± 0.14	1.67 ± 0.01	16.7 ± 0.4	11.3 ± 0.2	14.1 ± 0.5

For both the protein–ligand complex and solvated ligand steps, there was not a significant difference between Δ*G*s obtained from any of the short-run protocols and any of the long-run equilibration methods. While the restraint addition step did show significant deviation, the overall magnitude is small, and the errors are canceled on average in the other alchemical steps. However, the computational cost did vary across short-run protocols, with protocol C converging in approximately 50–60% of the simulation time allocated to protocol A depending on the alchemical step, with protocol B falling between the two but closer to C.

## Conclusions

We presented a data-driven procedure for the optimization of computational resource usage for both ABFE and RBFE calculations with thermodynamic integration. Our protocol is generally applicable to other free energy computational techniques that utilize stratified time series analysis. Our RBFE scheme affords up to 85% computational resource reduction compared to the CDK2 benchmark system results published by Song et al. while maintaining an average MAE of approximately 1 kcal/mol. Our protocols have successfully approximated long-run simulations of small RBFE mutations performed on the PLpro system, with the larger ligand 4 mutation deviating more significantly but still within 1 kcal/mol on average. Our ABFE schemes yield fast one-off calculations with similar accuracy compared to a base case of uniform and constant resource allocation on the T4 lysozyme L99A/M102Q mutant in complex with *N*-phenylglycinonitrile, and several implementations become more accurate while maintaining computational efficiency as the batch size increases. ABFE PLpro simulations displayed strong agreement between long-run 100 ns simulations and short-run simulations, with no significant deviation observed in the protein–ligand complex step, solvated ligand step, or overall computed Δ*G*_bind_^o^.

For future high-throughput RBFE campaigns, we recommend dividing mutations into two groups: “easy” mutations, consisting of those with few changes in heavy atoms or rotatable bonds, and “difficult” mutations, consisting of those with many changes in heavy atoms and rotatable bonds. For the easy mutations, we recommend performing one-off simulations using very short protocols (protocol C or D), as these have been shown to be just as accurate on both the CDK2 and PLpro systems while achieving significant computational savings. For more difficult mutations, longer protocols are appropriate (protocol A or E), as these require more sampling time to account for the larger amount of phase space available to the ligand. For future high-throughput ABFE campaigns, we recommend the utilization of protocol B as the best compromise between cost and accuracy. While protocol C or D, or even shorter protocols, may be employed to further reduce cost, we caution that prior testing should be performed to ensure that the requisite level of accuracy is maintained. The use of multiple replicates should be utilized to increase accuracy if resources allow it. In general, savings can be achieved by limiting the production simulation time of the solvated ligand and restraint addition steps, whereas resources should be concentrated on the protein–ligand complex step. This could be achieved by employing a mixed protocol utilizing protocol A or B for the protein–ligand complex and protocol C or D for the solvated ligand and restraint addition.

In this work, we benchmarked two hyperparameters of our workflow: the initial and additional simulation lengths. Future work should explore the role of the Jensen–Shannon distance convergence threshold, the maximum simulation lengths, the number of bins per histogram, and the number of requisite decorrelated samples. By adjusting the convergence threshold, one may be able to further optimize these protocols for efficiency or accuracy. In cases where significant reorganization of the protein occurs upon ligand annihilation, it may be necessary to increase the maximum simulation lengths or integrate more advanced sampling methods, such as Hamiltonian exchange molecular dynamics, into the workflow. The development of an on-the-fly optimal λ-schedule would further assist in increasing the efficiency of these calculations; however, this specific problem is beyond the scope of this work and will be addressed in the future. Finally, we note that our optimization algorithm is modular, and users may replace the automated convergence testing scheme with one they choose.

## Supplementary Material



## Data Availability

Scripts for the entire RBFE and ABFE workflows, as well as starting input, parameter, and topology files for all systems examined, are publicly available at https://github.com/MGKurnikovaGroup/otf_general_public.

## Notes

Description of the algorithm for the selection of virtual bond atoms; average MAE, RMSE, and computational savings by the alchemical step of RBFE simulations for the CDK2 benchmark system; representative RMSD plots for a long-run PLpro simulation; plots of average Δ*G*_bind_^o^ and its components for PLpro ligands 2 and 3 calculated from truncated trajectories; plots of d*V*/dλ convergency and dihedral angles distribution for PLpro ligand 4; and average total simulation time of ABFE simulations for lysozyme and PLpro systems (PDF)
