# Supplementary material for: Automated On-the-Fly Optimization of Resource Allocation for Efficient Free Energy Simulations
Source: J Chem Inf Model. 2025 May 6;65(10):4932–51. doi: 10.1021/acs.jcim.4c02107 (PMC12121625; doi:10.1021/acs.jcim.4c02107)
Supplement: Supplementary file 1 [file ci4c02107_si_001.pdf]

Supporting Information

An Automated On-The-Fly Optimization of Resource Allocation for High-Throughput Protein-Ligand Binding Free Energy Simulations

S. Benjamin Koby, Evgeny Gutkin, Shree Patel and Maria G Kurnikova

Department of Chemistry, Carnegie Mellon University, Pittsburgh, Pennsylvania, 15213, United States

Selection of virtual bond atoms for T4 Lysozyme L99A/M102Q.

Selection of atoms for the virtual bond approach[53] was performed using a modified version of the algorithm described in Chen *et al.,*[29] and proceeded as follows: All hydrogen bonding interactions present in 50% or more of the frames were selected and ranked based on the ligand atom distance from the ligand centroid. The atom closest to the centroid was then selected. If the ligand atom was a hydrogen, then the heavy atom bonded to this hydrogen was used instead. All neighboring heavy atoms and their corresponding neighboring heavy atoms were then selected. Protein and ligand atoms were scored based on their angles within the reference frame. Given protein backbone atoms A, B, and C, and ligand atoms a, b, and c, the following angles were calculated: ABC, aAB, baA, and abc (see Fig. S1). Atom combinations in which both angles were between 45° and 135° were considered valid. The possible ligand atom combinations fell into three categories. Within the first category, atom a was a heavy atom involved in hydrogen bonding, atom b a neighboring heavy atom of atom a, and atom c a neighboring heavy atom of atom b. Within the second category, atom a was the heavy atom involved in hydrogen bonding, atom b was a neighboring heavy atom of a neighboring atom of atom a, and atom c was a second neighboring heavy atom of the same neighboring heavy atom of atom a. In the third category, atoms a and c of category two are swapped with each other. For each of these categories, two combinations of protein heavy atoms of residue involved in hydrogen bonding were attempted: N-C_α-C and C- C_α-N. All valid configurations were then scored based on their mean absolute deviation from 90°, and the combination with the lowest score was selected for the virtual bond. If no valid atom combination was found, the next closest ligand atom to the centroid involved in hydrogen bonding was selected and analyzed for valid combinations. This process was repeated until a valid combination was found. If no atom combination was found to be valid, all ligand

heavy atoms were ranked based on their distance to the centroid of the ligand. The atom closest to the centroid was selected and compared against every combination of neighboring C_α atoms in the manner described above. If a valid combination was found, then the distance between ligand atom a and protein atom A was calculated. If the distance was found to be less than 1 nm, the combination was accepted as valid. If not, the next closest atom to the centroid was selected, and this process was repeated until a valid combination was found.

Table 1. Average MAE, RMSE, and Computational Savings by Alchemical Step of RBFE Simulations using Protocols A, B, and C of the CDK2 Benchmark System.

Simulation Protocol	*Batch Size*	*MAE (kcal/mol)*	*RMSE (kcal/mol)*	R^2	*Protein-Ligand Savings (%)*	*Solvated Ligand Savings (%)*
A	1	1.10 ± 0.09	1.35 ± 0.09	0.23 ± .06	75.6 ± 0.1	80.7 ± 0.0
B	1	1.13 ± 0.08	1.39 ± 0.08	0.20 ± .04	81.1 ± 0.2	87.0 ± 0.1
C	1	1.09 ± 0.09	1.37 ± 0.10	0.25 ± .06	85.6 ± 0.1	90.4 ± 0.1
C12	1	1.05 ± 0.08	1.33 ± 0.09	0.27 ± .05	64.1 ± 0.3	77.8 ± 0.1
A	2	1.06 ± 0.07	1.29 ± 0.06	0.25 ± .04	51.2 ± 0.2	61.4 ± 0.0
B	2	1.08 ± 0.06	1.33 ± 0.05	0.22 ± .03	62.1 ± 0.2	74.0 ± 0.1
C	2	1.04 ± 0.06	1.30 ± 0.07	0.26 ± .04	71.1 ± 0.2	80.7 ± 0.1
C12	2	1.00 ± 0.06	1.28 ± 0.06	0.29 ± .04	28.1 ± 0.4	55.6 ± 0.1
A	3	1.05 ± 0.05	1.27 ± 0.05	0.25 ± .03	26.7 ± 0.2	42.0 ± 0.1
B	3	1.05 ± 0.05	1.32 ± 0.04	0.22 ± .02	43.2 ± 0.2	60.9 ± 0.1
C	3	1.02 ± 0.05	1.28 ± 0.05	0.27 ± .03	56.7 ± 0.2	71.1 ± 0.1
C12	3	0.98 ± 0.05	1.26 ± 0.05	0.29 ± .03	-7.80 ± 0.50	33.5 ± 0.1
A	5	1.04 ± 0.03	1.25 ± 0.03	0.26 ± .02	-22.1 ± 0.2	3.40 ± 0.05
B	5	1.04 ± 0.03	1.30 ± 0.03	0.23 ± .02	5.28 ± 0.27	34.9 ± 0.1
C	5	1.00 ± 0.03	1.26 ± 0.03	0.28 ± .02	27.8 ± 0.2	51.8 ± 0.1
C12	5	0.97 ± 0.03	1.25 ± 0.03	0.30 ± .02	-79.7 ± 0.5	-11.0 ± 0.1

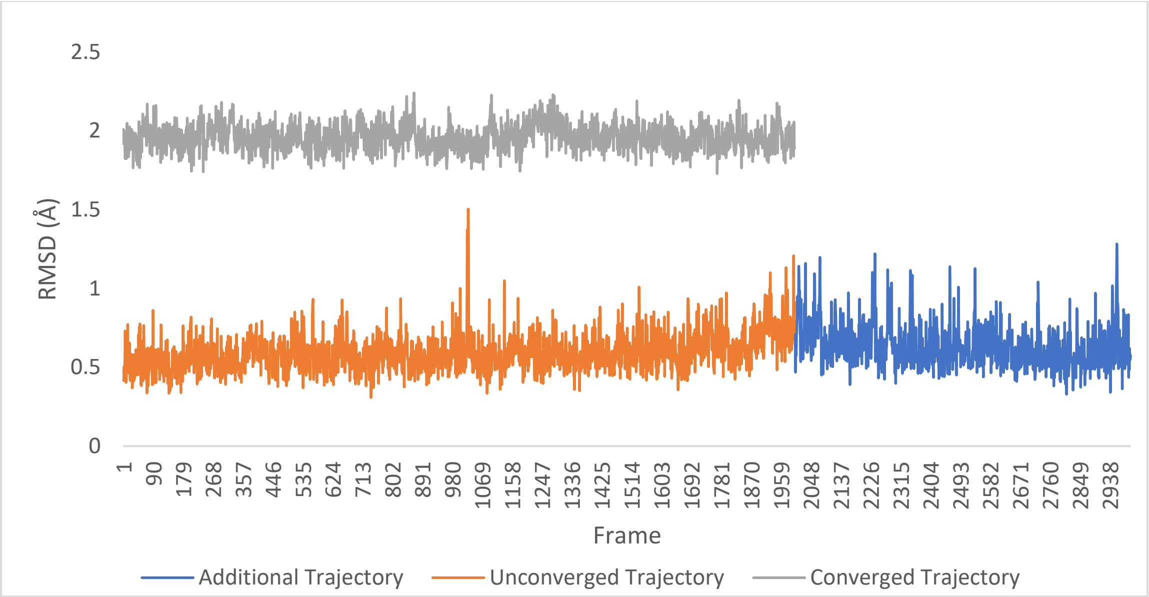

Figure S1. Representative RMSD plots of both ligand heavy atoms and binding pocket C_α atoms of a long-run PLpro simulation. If the initial 10 ns trajectory was not found to have converged, it was extended by 5 ns and convergence checked again.

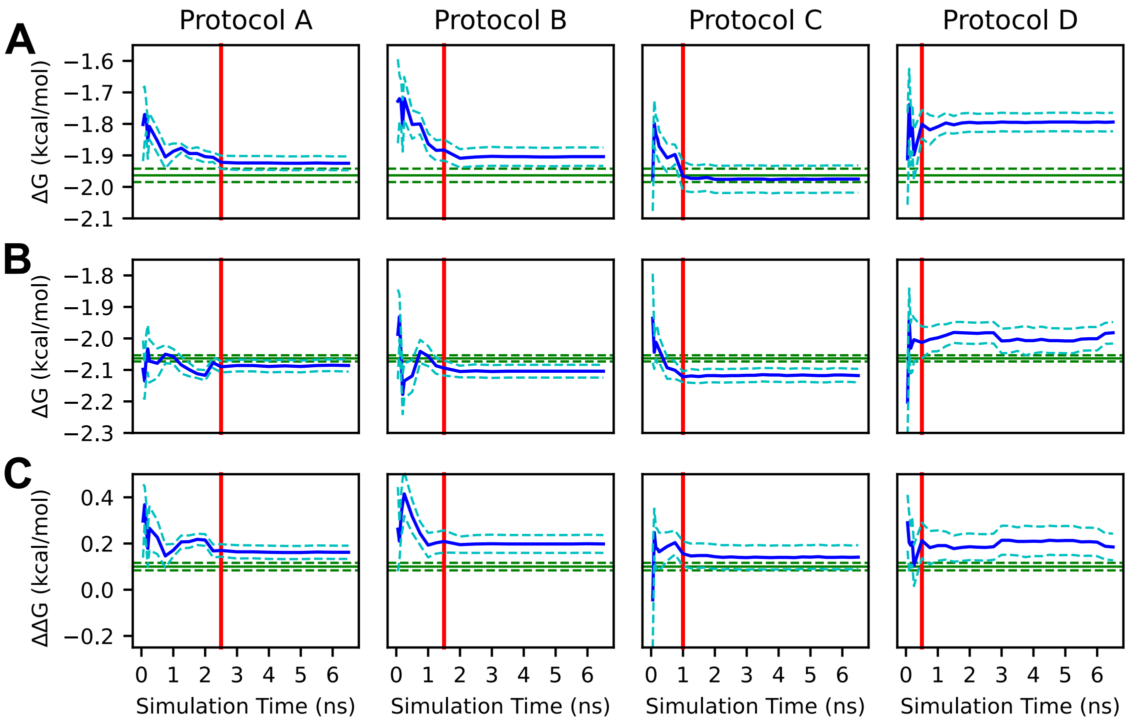

Figure S2. A) Average Ligand 2 protein-ligand complex ΔGs calculated from truncated gradients. B) Average Ligand 2 solvated ligand ΔGs calculated from truncated gradients. C) Average Ligand 2 $\Delta\Delta G^{bind}_{ref\to 2}$calculated from truncated trajectories. The blue line represents the mean ΔG or $\Delta\Delta G^{bind}_{ref\to 2}$ and

the blue dashed line represents one standard error of the mean. The green line represents the mean long-run ΔG or $\Delta\Delta G_{ref\rightarrow 2}^{bind}$ calculated using AED, the green dashed line represents one standard error of the mean, and the red line denotes the length of the initial simulation.

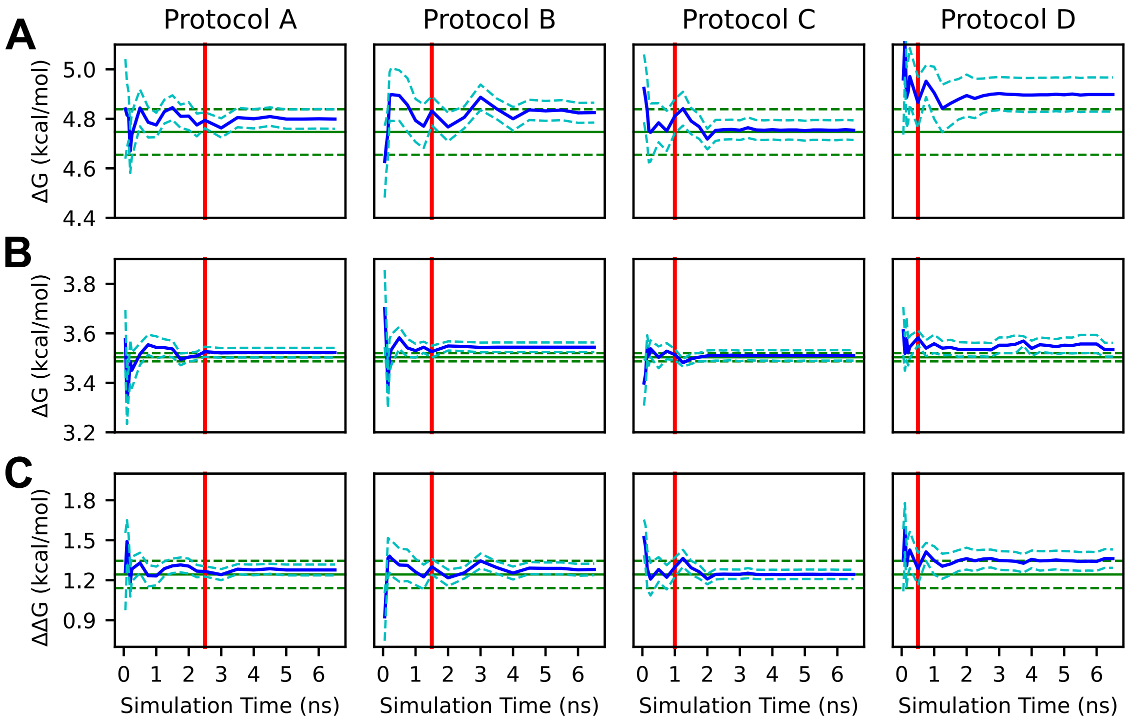

Figure S3. A) Average Ligand 3 protein-ligand complex ΔGs calculated from truncated gradients. B) Average Ligand 3 solvated ligand ΔGs calculated from truncated gradients. C) Average Ligand 3 $\Delta\Delta G_{ref\rightarrow 3}^{bind}$ calculated from truncated trajectories. The blue line represents the mean ΔG or $\Delta\Delta G_{ref\rightarrow 3}^{bind}$ and the blue dashed line represents one standard error of the mean. The green line represents the mean long-run ΔG or $\Delta\Delta G_{ref\rightarrow 3}^{bind}$ calculated using AED, the green dashed line represents one standard error of the mean, and the red line denotes the length of the initial simulation.

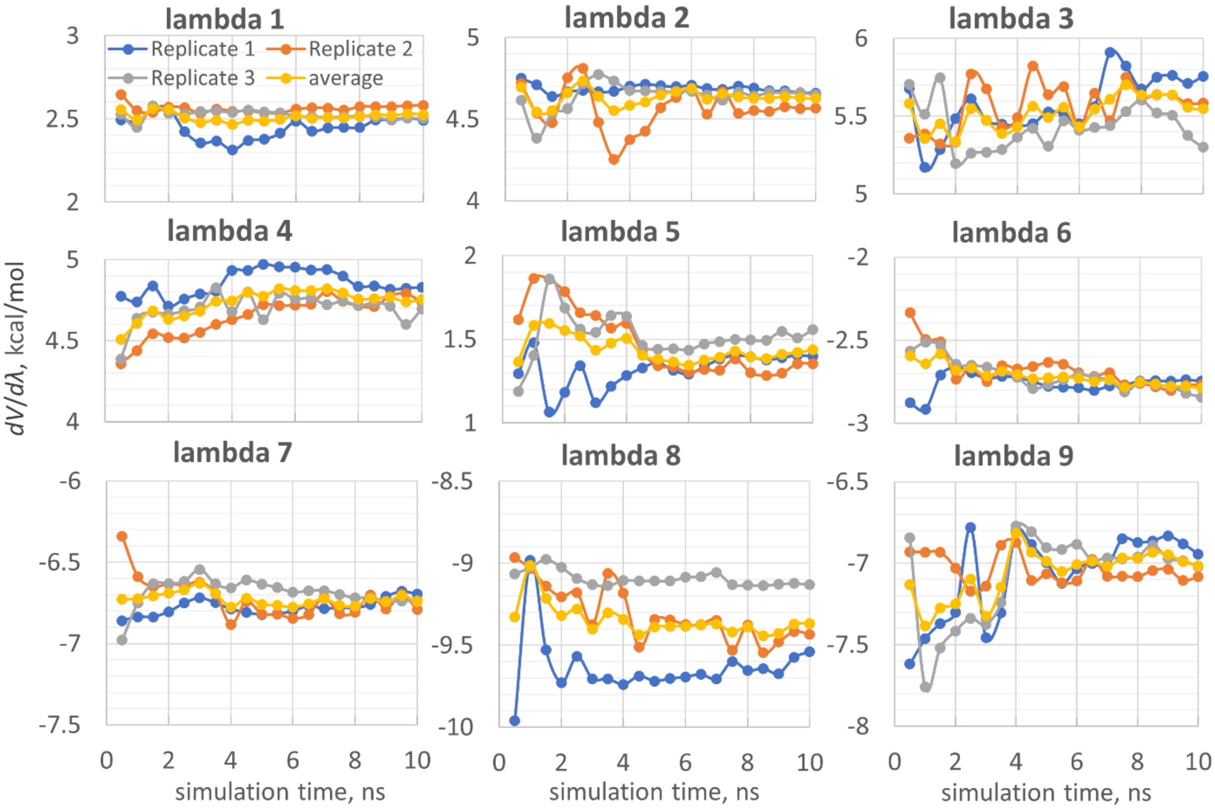

Figure S4. Convergency of *dV/dλ* for each lambda of protein-ligand complex step for PLpro Ligand 4.

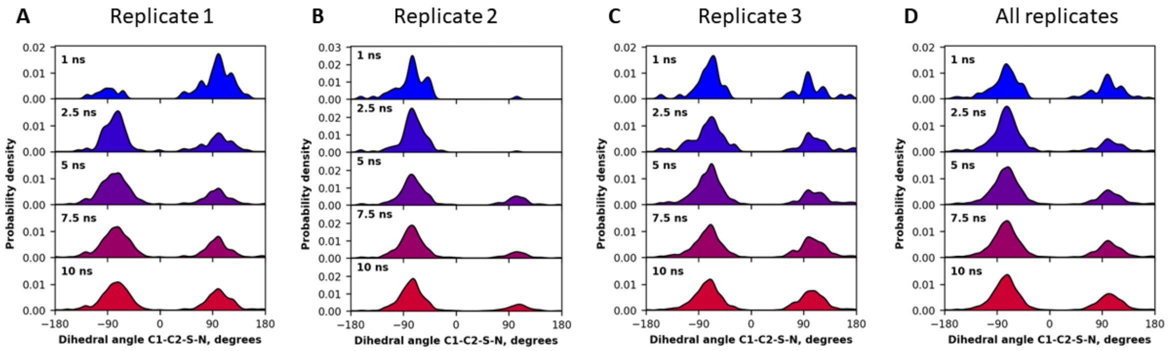

Figure S5. Distribution of dihedral angle C1-C2-S-N for PLpro ligand 4 for protein-ligand complex step production simulation at λ=0.5 for replicate 1-3 (A-C) and all replicates (D) at different simulation times.

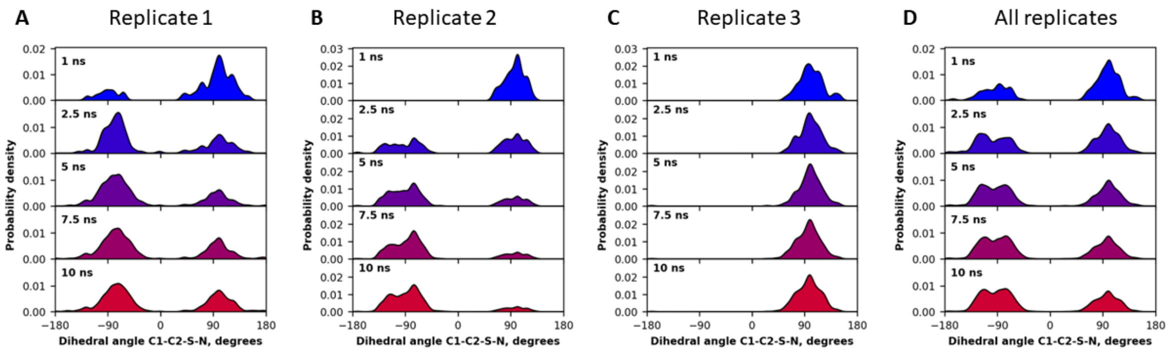

Figure S6. Distribution of dihedral angle C1-C2-S-N for PLpro ligand 4 for protein-ligand complex step production simulation at λ=0.8 for replicate 1-3 (A-C) and all replicates (D) at different simulation times.

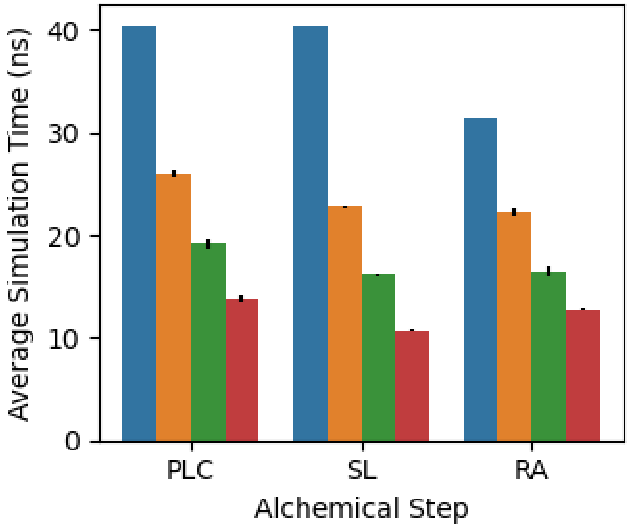

Figure S7. Average total simulation time of Lysozyme ABFE simulations by alchemical step and protocol. Protocol O is represented in blue, Protocol A in orange, Protocol B in green, and Protocol C in red. Note that error bars represent one standard error.

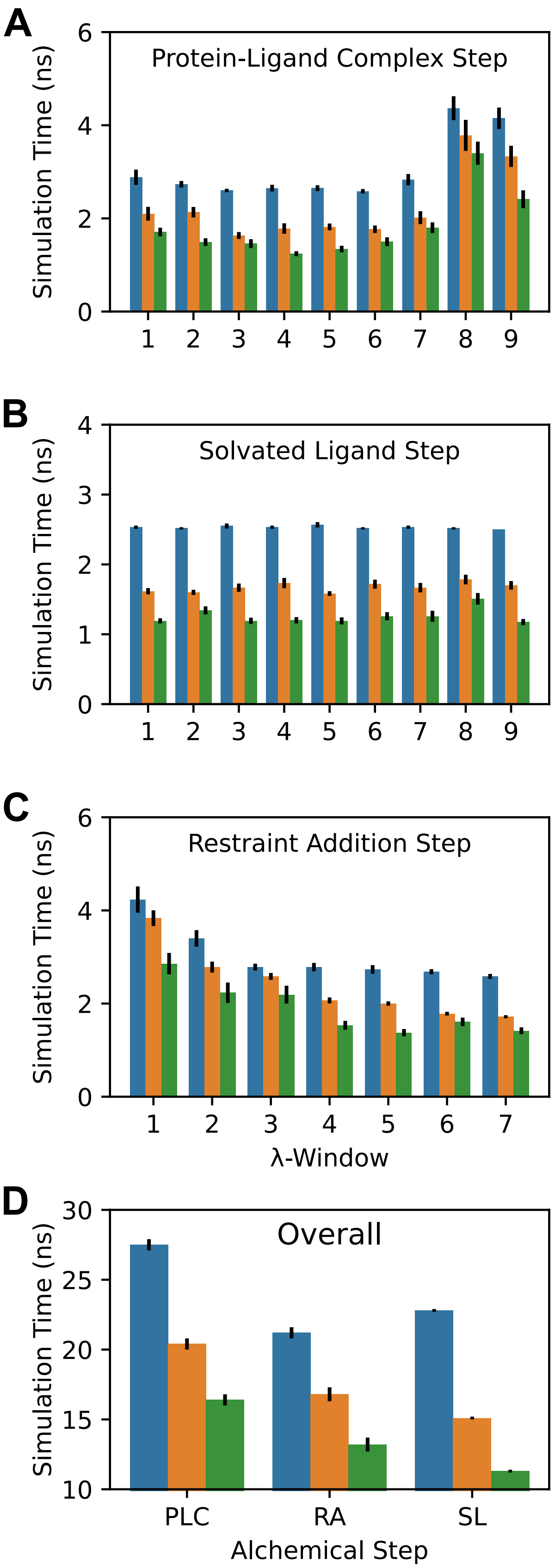
A
Protein-Ligand Complex Step
Simulation Time (ns)
B
Solvated Ligand Step
Simulation Time (ns)
C
Restraint Addition Step
Simulation Time (ns)
λ-Window
D
Overall
Simulation Time (ns)
PLC
RA
SL
Alchemical Step

Figure S8. A) Average simulation times of PLpro ABFE protein-ligand complex step simulations by λ-window and simulation protocol. B) Average simulation times of PLpro ABFE solvated ligand step simulations by λ-window and simulation protocol. C) Average simulation times of PLpro ABFE restraint addition step simulations by λ-window and simulation protocol. D) Average total simulation time of PLpro ABFE simulations per replicate by alchemical step and protocol. Blue depicts Protocol A, orange depicts Protocol B, and green depicts Protocol C. Error bars represent one standard error.
